# Effects of inoculation with active microorganisms derived from adult goats on growth performance, gut microbiota and serum metabolome in newborn lambs

**DOI:** 10.3389/fmicb.2023.1128271

**Published:** 2023-02-13

**Authors:** Lin Fu, Liaochuan Wang, Li Liu, Li Zhang, Ziyao Zhou, Yan Zhou, Gaofu Wang, Juan J. Loor, Peng Zhou, Xianwen Dong

**Affiliations:** ^1^Chongqing Academy of Animal Sciences, Chongqing, China; ^2^The Key Laboratory of Animal Disease and Human Health of Sichuan Province, College of Veterinary Medicine, Sichuan Agricultural University, Chengdu, China; ^3^Chongqing Chemical Industry Vocational College, Chongqing, China; ^4^Mammalian NutriPhysioGenomics, Department of Animal Sciences and Division of Nutritional Sciences, University of Illinois, Urbana, IL, United States

**Keywords:** active microorganism, inoculation with rumen fluid, newborn lambs, gut microbiota, blood metabolomics

## Abstract

This study evaluated the effects of inoculation with adult goat ruminal fluid on growth, health, gut microbiota and serum metabolism in lambs during the first 15 days of life. Twenty four Youzhou dark newborn lambs were selected and randomly distributed across 3 treatments (*n* = 8/group): autoclaved goat milk inoculated with 20 mL sterilized normal saline (CON), autoclaved goat milk inoculated with 20 mL fresh ruminal fluid (RF) and autoclaved goat milk inoculated with 20 mL autoclaved ruminal fluid (ARF). Results showed that RF inoculation was more effective at promoting recovery of body weight. Compared with CON, greater serum concentrations of ALP, CHOL, HDL and LAC in the RF group suggested a better health status in lambs. The relative abundance of *Akkermansia* and *Escherichia-Shigella* in gut was lower in the RF group, whereas the relative abundance of *Rikenellaceae_RC9_gut_group* tended to increase. Metabolomics analysis shown that RF stimulated the metabolism of bile acids, small peptides, fatty acids and Trimethylamine-N-Oxide, which were found the correlation relationship with gut microorganisms. Overall, our study demonstrated that ruminal fluid inoculation with active microorganisms had a beneficial impact on growth, health and overall metabolism partly through modulating the gut microbial community.

## 1. Introduction

The proper management of the pre-ruminant animal is important for ensuring they are able to face the weaning and early post-weaning periods without experiencing excessive stress. This represents one important pillar of a successful cattle and small ruminant industry. Intestinal microorganisms are important for regulating the health and development in the pre-wean ruminant animal. For example, intestinal bacterial imbalance is associated with disordered nutrient digestion and absorption, growth retardation, diarrhea, dehydration, and death (Signorini et al., 2012; Lyoo et al., 2018). Therefore, research focused on the establishment of an optimal intestinal microbiota would be beneficial in terms of promoting the development and health of pre-wean ruminants.

The microflora in ruminants is not fully developed at the moment of birth (Baldwin et al., 2004). Microbial colonization in pre-ruminans is divided into three stages: (1) the first 2–3 days of life are the initial colonization stage of “pioneer” bacteria, characterized by a unique profile relative to later stages of growth (Haenel, 2010). A large proportion of the bacterial community at this stage is parthenogenic and exclusively anaerobic but aerobic bacteria are also present (Minato et al., 1992); (2) the transition from colostrum to mature milk or milk replacer during which the ruminal epithelium microbial community and the ruminal environment change significantly with a dramatic decrease in aerobic and parthenogenic anaerobic bacteria (Rey et al., 2012); and (3) from day 14 to day 28 or at weaning, where bacterial communities no longer exhibit significant time-related changes, but rather change based on feeding behavior and growth (Meale et al., 2016). Bacterial species and abundance stabilize at the phylum level by the 15 day of age, and the bacterial community structure reaches a certain level of stability in about 3–4 weeks (Guzman et al., 2015). The ruminal microbiota digest fodder and other plant materials to produce volatile fatty acids (VFAs) and microbial crude protein (MCP), both of which provide energy and amino acids for ruminants (Koike and Kobayashi, 2009; Mccann et al., 2014). These are essential for maintenance of health, digestive efficiency, and growth performance in the growing ruminant animal (Morrison et al., 2010). Therefore, the first 15 days after birth are an important stage where the establishment of microbial flora can be regulated.

In a previous study, inoculation with a ruminal fluid increased the weight of calves during weaning partly due to a microbial profile that enhanced starch digestion (Ziolecki et al., 1984). Direct-fed microorganisms were reported to improve immunity of calves by balancing gut microbe profiles, thus, helping them cope with stressful conditions (Krehbiel et al., 2003). Mechanistically, the gut microbiota can stimulate immune responses through its effect on digestion and metabolism (Anand and Mande, 2018). For example, it has been demonstrated that microbiota can regulate metabolism of glutamate and creatine, which in turn impacts the immune response (Ding et al., 2021). Therefore, accumulating evidence highlights that the establishment of the microflora is important in newborn ruminants and can influence their metabolism.

Some studies reported that the original composition of the rumen returned to a pre-intervention state after exogenous microbiota interventions were discontinued (Weimer, 2015). In spite of this, there are data indicating that exogenous microbiota transplantation could improve ruminal fermentation and digestion. To our knowledge, few studies have focused on the effect of ruminal microbial transplantation within 15 days after birth on development and health (Jami et al., 2013). Thus, we hypothesized that inoculation with ruminal fluid in newborn lambs could promote growth and health through its effect on the establishment of an “ideal” gut microbiota. Such benefits are reflected in blood metabolite profiles. To address this hypothesis, Youzhou dark newborn lambs were inoculated with sterilized normal saline (CON), fresh ruminal fluid (RF) with active microorganisms or autoclaved ruminal fluid (ARF) without active microorganisms. Growth performance, health status, gut microbiota and blood metabolomics were performed to evaluate treatment effects during the first 15 days after birth.

## 2. Materials and methods

The study procedures and use animals were approved by the Ethics Committee in Chongqing Academy of animal sciences (approval number: xky-20180716, 9 June 2021).

### 2.1. Ruminal inoculum preparation

Six healthy Youzhou dark adult goats (three female and three male, ~30 kg) were used as sources of ruminal fluid. All goats were fed with the same diet for 3 weeks prior to ruminal fluid collection ~2 h post-feeding *via* oral tubing, strained into a container through four layers of cheesecloth and then pooled into a composited sample. All the composited ruminal fluid was divide into two samples. One sample was directly stored at −80°C and served as the “fresh” ruminal fluid (RF). The other portion of sample was sterilized at 205.8 KPa and 132°C for 10 min and served as the autoclaved Ruminal Fluid (ARF), which was subsequently stored at −80°C. All original samples were kept at −80°C until use as the inoculum.

### 2.2. Animals and treatments

A total of 24 Youzhou dark newborn lambs were selected and randomly assigned to one of 3 treatments for a feeding period of 15 days in individual pens. During the initial 48 h after birth, all lambs received colostrum from their mothers, and were then separated from their mothers immediately. Treatments consisted of (1) sterilized goat milk inoculated with sterilized normal saline as the control group (CON); (2) sterilized goat milk inoculated with RF (RF) and (3) sterilized goat milk inoculated with ARF (ARF). Sterilized goat milk was fed 4 times (each time ~100 mL) daily (0800, 1200, 1600 and 2000 h) to ensure that all lambs had adequate nutrition. From day 3 to 7 after birth, 20 mL ruminal fluid or autoclaved ruminal fluid was maintained at 39°C in a prewarmed thermostat water bath. It was then inoculated *via* the esophagus of each lamb using a soft stomach tube and a 20 mL syringe 2 h after the morning feeding (inoculate once a day, a total of 20 mL). The experiment lasted 15 days, and the all lambs were slaughtered on day 16.

### 2.3. Growth performance and sampling

The live weight (LW) of each lamb was recorded every 3 days before the morning feeding during the experimental period (15 days). Average daily gain (ADG) was calculated as (final LW–initial LW)/days on study. On the 16th day of the experimental period, a blood sample (~5 mL) was collected from the jugular vein and then placed into blood collection tubes. Blood samples were centrifuged at 2,500 rpm at 4°C for 15 min to obtain serum and then stored at −80°C until analysis. Fecal samples from each lamb were collected on the 16th day of the experimental period and stored at −80°C until gut microbiota profiling.

### 2.4. Sample analyses

#### 2.4.1. Blood biochemical indices determination

An automatic biochemical analyzer (Beckman Coulter AU680) was used to perform blood biochemical analysis. Briefly, assays included colorimetric kits (modified kinetic Jaffe method), turbidimetry, latex agglutination, homogeneous EIA or indirect ISE according to the Beckman Coulter AU680 analyzer specifications.

#### 2.4.2. 16S rRNA sequencing of gut microbiota

Total DNA was extracted from 1 g feces using the MoBio PowerSoil DNA Isolation Kit (12855-50, MoBio, United States) according to the manufacturer’s instructions. The quantity and quality of DNA were measured using NanoDrop 2000 spectrophotometer (Thermo Fisher Scientific, United States) Then DNA integrity was determined with 1% agarose gel electrophoresis.

Subsequently, the V3-V4 hypervariable region of the 16S rRNA gene was PCR-amplified with the universal primer pair 338F (5′-ACTCCTACGGGAGGCAGCAG-3′) and 806R (5′-GGACTACHVGGGTWTCTAAT-3′) (Liu et al., 2022). The PCR amplification was based on the protocol of Chan et al. (2015). Briefly, 10 bp barcode sequence was added to the 5′ end of the forward and reverse primers (provided by Allwegene Company, Beijing). PCR amplification was performed with a 25 μL reaction system including 12.5 μL Taq PCR MasterMix (2×), 3 μL BSA (2 ng/μL), 1 μL (5 μM) forward primer, 1 μL (5 μM) reverse primer, 2 μL dDNA (The total amount of DNA added is 30 ng) and 5.5 μL ddH_2_O. Thermal cycling parameters were as follows: 95°C for 5 min, 28 cycles of 95°C for 45 s, 55°C for 50 s, 72°C for 45 s and final extension 72°C for 10 min. Purification of PCR products was carried out using Agencourt AMPure XP Kit (Beckman, Brea, CA, United States). Real-time PCR was used for PCR product quantification. Deep sequencing was carried out using IllluminaMiSeq PE300 platform at Allwegene Company (Beijing, China). Image analysis, base calling, and error estimation were performed using Illumina Analysis Pipeline Version 2.6.

Read qualification was performed using Illumina Analysis Pipeline Version 2.6. The low-quality sequences with length < 230 bp, average Phred scores <20 and ambiguous bases or false matches to primer sequences and barcode tags were removed. High-quality sequences were clustered into operational taxonomic units (OTUs) at a similarity level of 97% using Uparse algorithm of Vsearch (v2.7.1) software. The Ribosomal Database Project (RDP) Classifier tool was used to conduct OTU taxonomic classification into different taxonomic groups against the SILVA128 database. The rarefaction curve generation and richness and diversity indices calculation were performed using QIIME (version 1.8.0) based on the OTU information.

### 2.5. Metabolite extraction

Metabolomics was performed at Allwegene Company (Beijing, China). Briefly, a total of 20 μL of sample was transferred to an Eppendorf tube. A volume of 80 μL extraction solvent was added (acetonitrile: methanol = 1: 1, containing isotopically-labeled internal standard mixture). The mixture was sonicated in an ice-water bath for 10 min and incubated at −40°C for 1 h to precipitate proteins. The samples were centrifuged at 4°C, 12,000*g* for 15 min, then supernatant fluid was transferred into a EP tube new glass vial for UHPLC–MS–MS analysis. QC samples were created by merging equal aliquots of supernatant fluid from each sample.

### 2.6. UHPLC-MS-MS analysis

UHPLC-MS-MS analysis was performed using a UHPLC system (Vanquish, Thermo Fisher Scientific) with a UPLC BEH amide column (2.1 mm 100 mm, 1.7 m) coupled to a Q Exactive HFX mass spectrometer (Orbitrap MS, Thermal). Mobile phase consisted of 25 mmol/L ammonium acetate and 25 mmol/L ammonia (pH = 9.75). The autosampler was set to 2 L injection volume at 4°C. The QE HFX mass spectrometer with the acquisition software information-dependent acquisition (IDA) mode (Xcalibur, Thermo) was used for obtaining MS/MS spectra. The ESI conditions were: Sheath gas flow of 30 Arb, auxiliary gas flow of 25 Arb, capillary temperature of 350°C, and full MS resolution of 60,000.

Metabolomics analysis was performed based on our previous studies with slight modifications (Dong et al., 2018; Fu et al., 2021). Briefly, principle component analysis (PCA) and (orthogonal) partial least-squares-discriminant analysis (OPLS-DA) were performed using the R package metaX to monitor the reproducibility of the instrument and the differential analysis of metabolic characteristics. Parameters R2Y and Q2 were >0.5 indicating a robust model with prominent predictive ability. Metabolites with variable importance for projection (VIP) values exceeding 1 and *p* < 0.05 were selected as the important metabolites between the comparison of two groups.

### 2.7. Statistical analysis

Statistical analysis of weight indices, blood biochemical indices and diversity and relative abundance of gut microbiota were performed using GraphPad Prism 7.0 (GraphPad Software). All data are presented as means ± SEM. The KS normality test was performed to estimate data normality. Statistical differences between groups was assessed using one-way analysis of variance (ANOVA), and then multiple comparisons analysis was performed with a Tukey *post-hoc* test. Data with non-normal distribution was analyzed using the Kruskal–Wallis test and then the Dunn’s multiple comparison test. *p* < 0.05 was considered as significant difference. The correlation between the gut microbiota and serum metabolites was analyzed using Spearman correlations with the R program package. The coefficients *p* < 0.05 were considered significant. The R language GGPlot package was used to draw a correlation heat map.

## 3. Results

### 3.1. Growth performance

**Figure 1:**
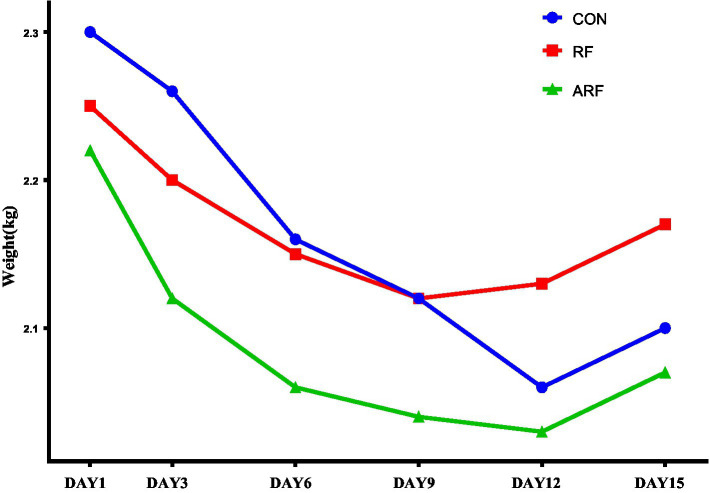
Trend of body weight change during postnatal 15 days in lambs inoculated with ruminal fluid (RF), autoclaved ruminal fluid (ARF) or sterilized physiological saline (CON).

The effects of inoculation with ruminal fluid (RF), autoclaved ruminal fluid (ARF) or sterilized physiological saline (CON) on growth performance of newborn lambs are presented in Figure 1 and Supplementary Table 1. The RF resulted in the lowest weight loss compared with CON and ARF. Moreover, for all groups the trend of weight change was to decrease first followed by an increase. Interesting, RF lambs stopped losing weigth on day 9 after birth, and then followed recovery. This was 3 days earlier than CON and ARF groups.

### 3.2. Blood biochemical indicators

The effects of inoculation with ruminal fluid (RF), autoclaved ruminal fluid (ARF) or sterilized physiological saline (CON) on blood biochemical indices are shown in Table 1. Compared with CON, feeding RF resulted in a greater (*p* < 0.05) blood concentration of ALP, CHOL, HDL, and LAC. Moreover, a greater (*p* < 0.05) blood concentration of ALP was also observed in RF relative to ARF. Compared with CON, ARF led to lower (*p* < 0.05) blood concentration of DBIL. No significant differences in concentrations of ALT, AST, GGT, AST/ALT, TAB, TP, ALB, GLO, A/G, TBIL, IDBIL, CHE, BUN, CREA, TG, LDL, CRP, GLU and LDH were observed among all groups.

**Table 1:** Effect of inoculation with ruminal fluid (RF), autoclaved ruminal fluid (ARF) or sterilized physiological saline (CON) on blood biochemical indices in lambs.

Item	CON	RF	ARF	SEM	Value of *p*
ALT (U/L)	9.60	11.25	10.50	0.764	0.1144
AST (U/L)	79.60	104.20	91.00	11.140	0.1077
GGT (U/L)	63.80	67.40	71.00	12.620	0.8388
ALP (U/L)	350.00^a^	580.50^b^	257.20^a^	77.620	0.0032
AST/ALT	8.00	10.00	9.75	1.548	0.3577
TBA (μmol/L)	8.10	15.68	15.26	4.289	0.1483
TP (g/L)	71.98	71.76	71.70	4.358	0.9974
ALB (g/L)	31.95	32.72	32.46	1.803	0.9090
GLO (g/L)	40.03	39.04	39.24	4.504	0.9700
A/G	0.82	0.88	0.84	0.130	0.8785
TBIL (μmol/L)	2.50	2.140	1.94	0.415	0.3823
DBIL (μmol/L)	1.02^a^	0.700^ab^	0.65^b^	0.144	0.0360
IDBIL (μmol/L)	1.48	1.56	1.28	0.322	0.6675
CHE (U/L)	183.30	177.00	185.60	7.414	0.5010
BUN (mmol/L)	11.21	13.34	12.88	1.232	0.1950
CREA (μmol/L)	49.53	51.26	63.36	7.196	0.1406
CHOL (mmol/L)	5.21^a^	7.16^b^	6.182^ab^	0.484	0.0038
TG (mmol/L)	0.16	0.27	0.23	0.046	0.0896
HDL (mmol/L)	1.99^a^	2.72^b^	2.45^ab^	0.265	0.0393
LDL (mmol/L)	2.82	3.81	4.07	0.601	0.1041
CRP (mg/L)	4.53	4.36	4.78	0.409	0.5874
GLU (mmol/L)	2.81	2.73	3.72	0.748	0.3566
LAC (mmol/L)	7.02^a^	10.33^b^	10.17^b^	0.727	0.0004
LDH (U/L)	394.30	439.00	339.50	39.200	0.0725

^a,b^Letters means within a row that do not have a common superscript letter differ, *p* < 0.05. ALT, glutamate pyruvic transaminase; AST, Aspartate aminotransferase; GGT, glutamyl transferase; ALP, alkaline phosphatase; TBA, Total bile acid; TP, Total protein; ALB, albumin; GLO, Globulin; T-Bil, total bilirubin; D-Bil, Direct bilirubin; IDBIL, Indirect bilirubin; CHE, cholinesterase; BUN, blood urea nitrogen; CREA, creatinine; CHOL, total cholesterol; TG, triglyceride; HDL-C, High density lipoprotein; LDL-C, Low density lipoprotein; CRP, C-reactive protein; GLU, glucose; LAC, lactate acid concentration; LDH, lactate dehydrogenase.

### 3.3. Gut microbiota

**Figure 2:**
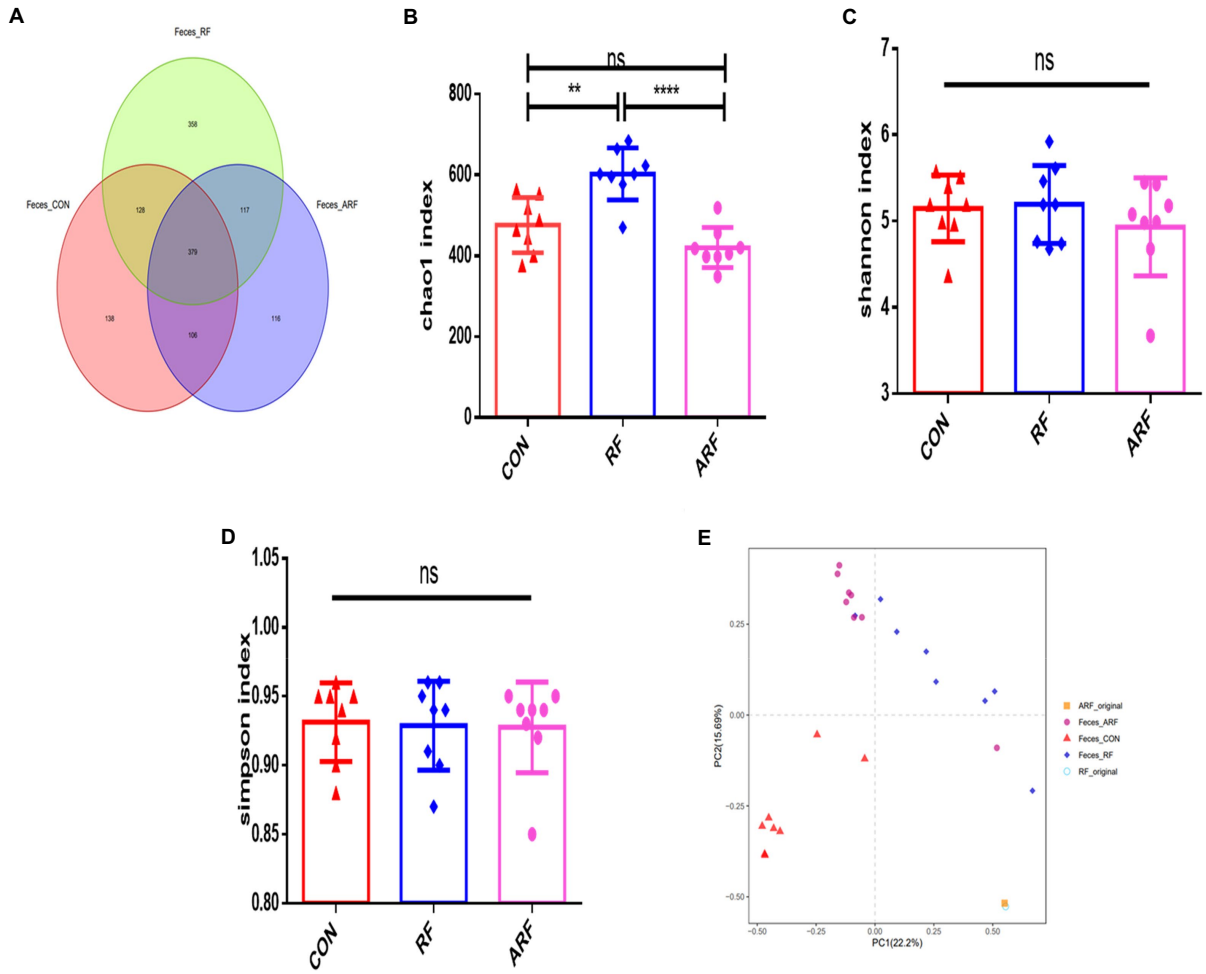
Gut Microbiota diversity analysis in 15-day old lambs after inoculation with ruminal fluid or autoclaved ruminal fluid. **(A)** Unique and shared intestinal operational taxonomic units (OTUs) for each group are shown in the Venn diagram. Alpha diversity (Chao1index, Shannon index, Simpson index) **(B–D)**; beta diversity indicated by principal component analysis (PCA) plot **(E)**. Data are presented as means ± SD. *n* = 8, **p < 0.01, ****p < 0.0001, ns indicates no significance.

Under the similarity threshold of 97%, 1,342 OTUs were obtained and the calculated good’s coverage were no <99% for all samples. Venn diagrams showed that the number of OTUs in the RF group was greater compared with other groups. (Figure 2A). The Chao1 index, Shannon index and Simpson index were used to estimate the Alpha diversity of gut microbiota. Compared with the other groups, the Chao1 index indicated that feeding RF substantially increased the heterogeneity of the gut microbiota (Figure 2B). However, no significant difference among the groups emerged in the Shannon index and Simpson index (Figures 2C,D). The PCA analysis was used to compare the microbial community composition and distribution similarity of each group. According to the distance and separation of each sample in each group in the figure, it is evident that the CON group was markedly different from those of the RF group, and there were differences between the CON group and the ARF group (Figure 2E). Interestingly, the microbial composition was significantly different from that of the original samples (RF-Original and ARF-Original), and the original samples were similar in microbial composition.

**Figure 3:**
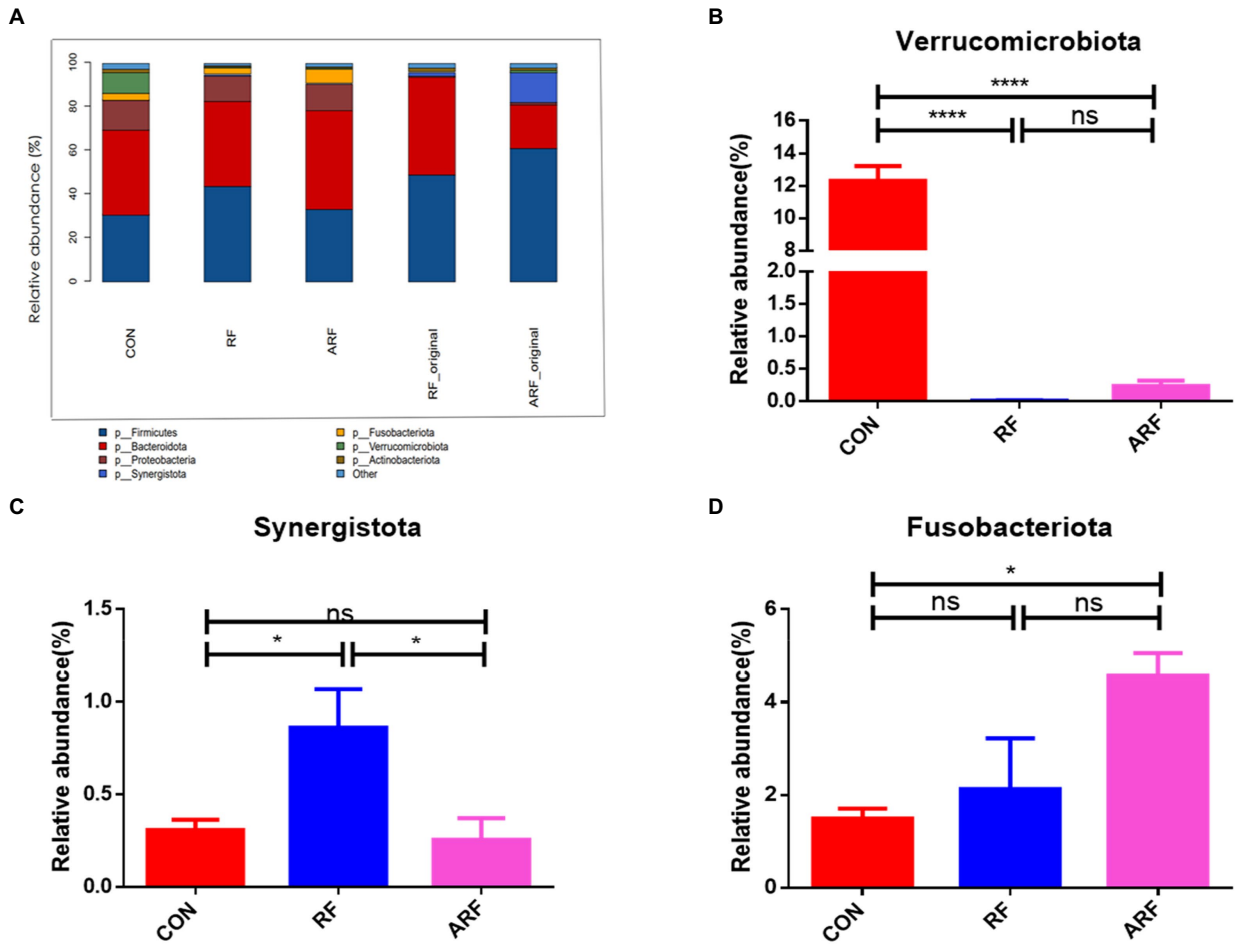
Relative abundance of microbiota at the phylum level. **(A)** Microbiota taxonomic profiling of intestinal microbiota from different groups at the phylum level with the abundance of the microbiota is more than 1%. **(B)** Ratio of *Verrucomicrobiota* in the three groups. **(C)** Ratio of *Synergistota* in the three groups. **(D)** Ratio of *Fusobacteriota* in the three groups. *n* = 8, *p < 0.05, **p < 0.01, ***p < 0.001, ****p < 0.0001, ns indicates no significance.

We then focused on microbiota abundance at the phylum and genus levels. At the phylum level, *Bacteroidetes*, *Firmicutes*, *Verrucomicrobiota* and *Proteobacteria* were the 4 most dominant bacterial phyla in the three groups (Figure 3A). The statistical analysis showed that, at the phylum level, the gut microbiota in three groups were dominated by *Firmicutes*, *Bacteroidota*, *Proteobacteria*, *Synergistota*, *Fusobacteriota*, *Verrucomicrobiota*, and *Actinobacteriota*, while the relative abundance of other genera was below 1% (Figure 3A). The statistical analysis of abundant genera (with relative abundance >0.01%) showed that the relative abundance of *Verrucomicrobiota* was significantly lower (*p* < 0.05), while the relative abundance of *Synergistota* was significantly higher (*p* < 0.05) in the RF group than those in the CON group (Figures 3B,C). The abundance of *Synergistota* in the RF group was higher than that in other groups (Figure 3C). More details about differential microbiota abundance are shown in Supplementary Figure 1. The relative abundance of *Fusobacteriota* in the ARF group was significantly higher than that in the CON group (Figure 3D).

**Figure 4:**
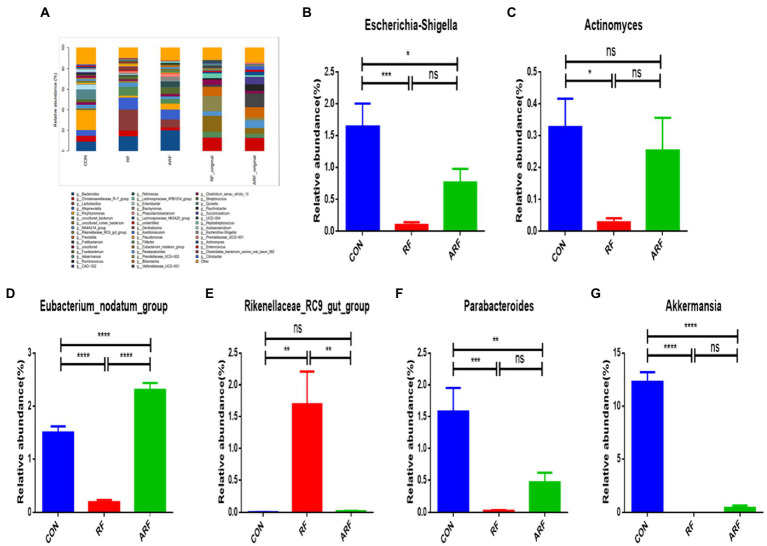
Relative abundance of microbiota at the genera level. **(A)** Microbiota taxonomic profiling of intestinal microbiota from different groups at the genera level which the abundance of the microbiota is more than 1%. **(B)** Ratio of *Escherichia-Shigella* in the three groups. **(C)** Ratio of *Actinomyces* in the three groups. **(D)** Ratio of *Eubacterium_nodatum_group* in the three groups. **(E)** Ratio of *Rikenellaceae_RC9_gut_group* in the three groups. **(F)** Ratio of *Parabacteroides* in the three groups. **(G)** Ratio of *Clostridiales_bacterium_canine_oral_taxon_082* in the three groups. **(H)** Ratio of *Akkermansia* in the three groups. *n* = 8, *p < 0.05, **p < 0.01, ***p < 0.001, ****p < 0.0001, ns indicates no significance.

The analysis of the relative abundance of the bacterial genera, 29 out of the 46 genus identified showed significant differences based on the inoculation treatment. *Bacteroides*, *Christensenellaceae_R−7_group*, *Porphyromonas* and *Alloprevotella* were the common genera dominated in the gut microbiota of the lambs (Figure 4A; Supplementary Figure 2). Our results revealed that 13 genera were significantly more predominant in the RF than CON (Figure 4E; Supplementary Figure 2). They included the *Rikenellaceae_RC9_gut_group*, *Bacteroides*, *Brachymonas*, *Peptostreptococcus*, *Petrimonas*, *Phascolarctobacterium*, *Pseudomonas*, and others. The relative abundance of *Actinomyces*, the *Escherichia-Shigella*, the *Eubacterium_nodatum_group*, *Parabacteroides* and *Akkermansia* was significantly lower (*p* < 0.05) in the RF group than those in the CON group (Figures 4B–G).

### 3.4. Serum metabolic profiles

**Figure 5:**
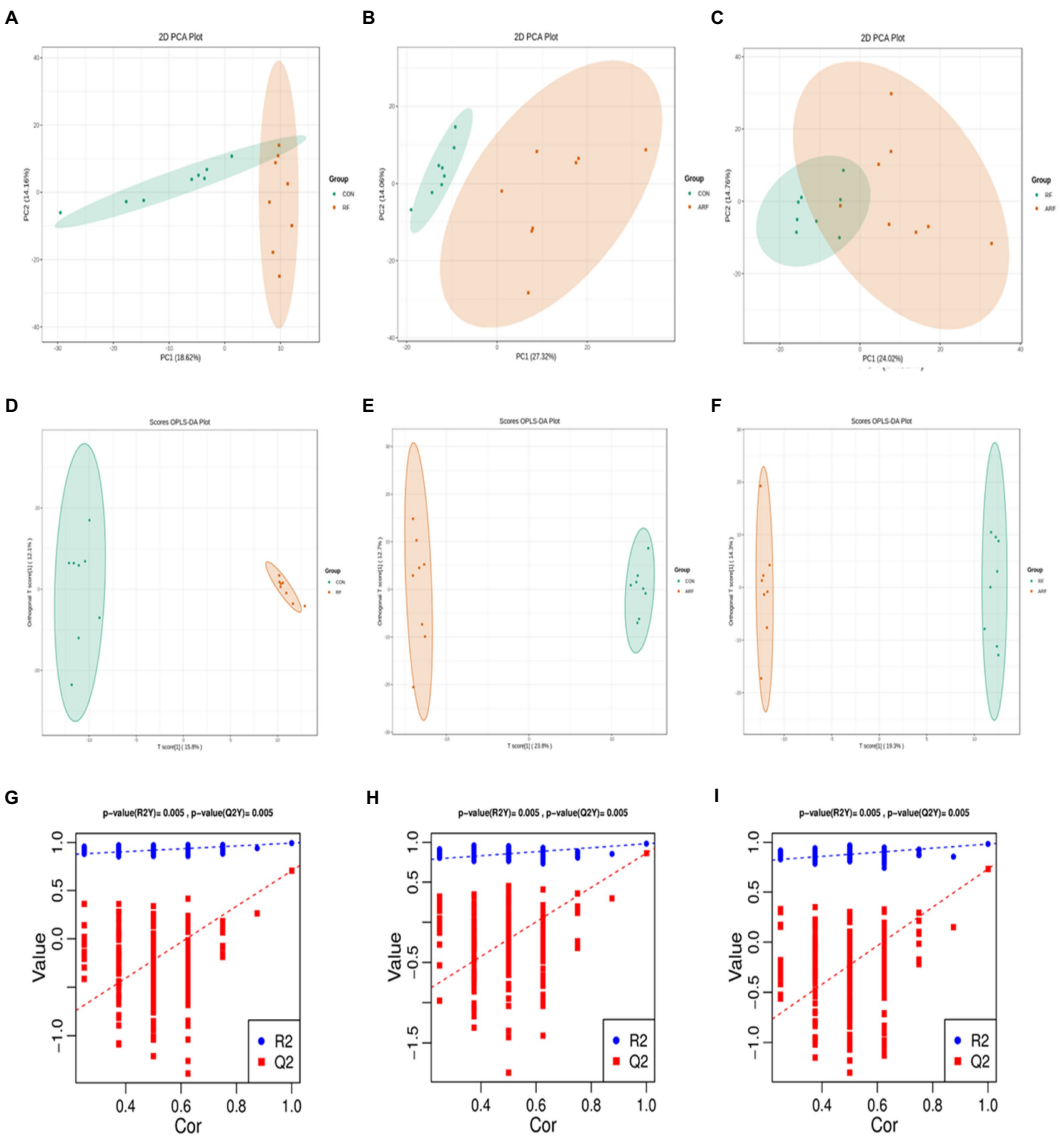
Metabolomics of PCA analysis, CON group (green) and RF group (red) **(A)**, OPLS-DA score chart, CON group (green) and ARF group (red) **(B)**, OPLS-DA score chart, RF group (green) and ARF group (red) **(C)**. OPLS-DA score chart, CON group (green) and RF group (red) **(D)**, OPLS-DA score chart, CON group (green) and ARF group (red) **(E)**, OPLS-DA score chart, RF group (green) and ARF group (red) **(F)**. Permutation test of OPLS-DA **(G–I)**.

According to the results obtained, a total of 807 metabolites were identified and quantified. All samples were analyzed with a 95% confidence interval. We performed PCA score plot to visualize the overall change of metabolites, the results showed differences between the three groups (Figures 5A–C). The OPLS-DA revealed a clear separation between the CON and the RF groups in the plot, which indicated that the serum metabolic profiles of the RF group were distinct from that of the CON group (Figure 5D). The other two figures also reflect similar results (Figures 5E,F). In the OPLS-DA model, the parameter R2Y was 0.998, and the Q2 value was 0.89 (Supplementary Figure 3), indicating a good degree of reliability and predictive ability of the model used. The OPLS-DA model involved 200 random permutations and combination experiments on the data to avoid over-fitting. A good model is obtained when all replacement models of R2 and Q2 values are lower than the original values of R2 and Q2. The study results suggested that the OPLS-DA model was not over fitted as demonstrated by the R2 and Q2 values in all permutated models being lower than the value of the original R2 and Q2 models (Figures 5G–I).

**Figure 6:**
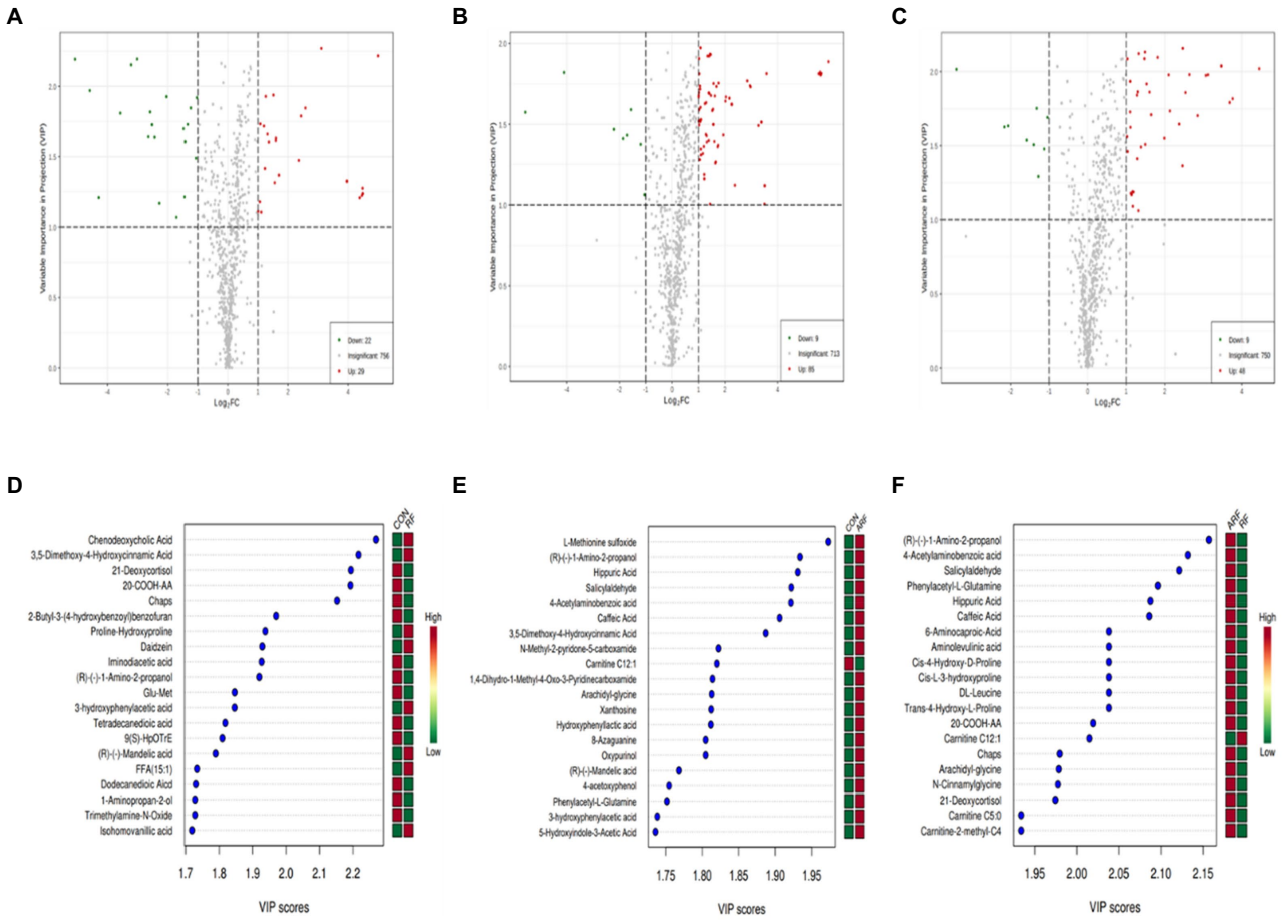
Differential metabolites in lamb serum inoculated with ruminal fluid. Volcano plots of the affected metabolites in CON *VS* RF **(A)**, CON *VS* ARF **(B)** and RF *VS* ARF **(C)**. Each point represents a metabolite. The green dots in the figure represent the down-regulated differential metabolites, the red dots represent the up-regulated differential metabolites, and the gray dots represent the metabolites without difference. Metabolites are ranked by variable importance in projection analysis (VIP) of respective groups: CON group and RF group **(D)**, CON group and ARF group **(E)** and RF group and ARF group **(F)**; the top 20 important metabolites were arranged from top to bottom according to intracellular concentration. The red box represents an up-regulated concentration of the molecule and the green box represents down-regulated concentration.

The results from the screening of different metabolites were visualized in the form of volcano plots to quickly assess the difference and statistical significance in the metabolic expression levels for the CON, RF and ARF group (Figures 6A–C). Fifty-one different metabolites were observed in CON vs. RF, including 22 decreased and 29 increased metabolites (Figure 6A); 94 different metabolites in CON vs. ARF, including 9 decreased and 85 increased metabolites (Figure 6B) and 57 different metabolites in RF vs. ARF, including 9 decreased and 48 increased metabolites (Figure 6C). The top 20 important differential metabolites with VIP > 1.5 and *p* < 0.05 are depicted in VIP plots (Figures 6D–F). Compared with CON, inoculation with ruminal fluid increased the plasma relative concentrations of Chenodeoxycholic Acid, Proline-Hydroxyproline, 3-hydroxyphenylacetic acid and FFA(15:1), whereas it decreased the plasma relative concentrations of 21-Deoxycortisol, 20-COOH-AA, Glu-Met, Tetradecanedioic acid, 9(S)-HpOTrE and Trimethylamine-N-Oxide (Figure 6D). Compared with CON, inoculated with autoclaved rumen fluid increased the plasma relative concentrations of Hippuric Acid, Caffeic Acid, Arachidyl-glycine, Xanthosine, Oxypurinol and 5-Hydroxyindole-3-Acetic Acid, whereas it decreased the plasma relative concentrations of Carnitine C12:1(Figure 6E). Compared with RF, inoculated with autoclaved rumen fluid increased the plasma relative concentrations of Phenylacetyl-L-Glutamine, DL-Leucine, Cis-L-3-hydroxyproline, 6-Aminocaproic-Acid, 20-COOH-AA, Caffeic Acid, Arachidyl-glycine, 21-Deoxycortisol and Carnitine C5:0, whereas it decreased the plasma relative concentrations of Carnitine C12:1(Figure 6F).More details about differential metabolites are shown in Supplementary Tables S1–S3.

### 3.5. Correlations between serum metabolic profiles and gut microbiota

**Figure 7:**
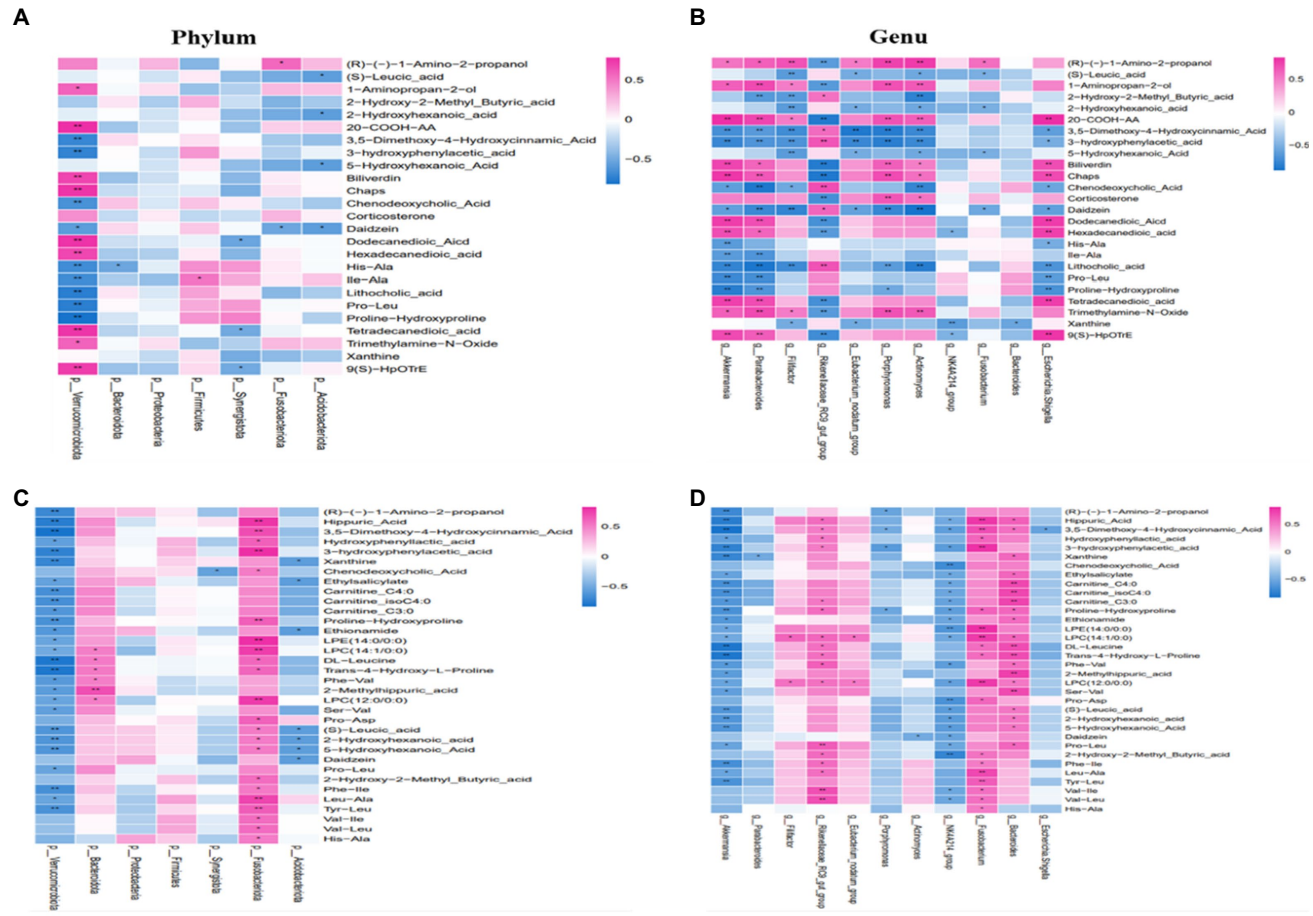
The correlation between gut microbiota and serum metabolites. At the phylum or genera level in the CON and RF groups **(A,B)**. At the phylum or genera level in the CON and ARF groups **(C,D)**. Red represents positive correlation, and green represents negative correlation. *p < 0.05, **p < 0.01.

Using the Spearman rank correlation coefficients, we displayed the results in the heatmap chart and evaluated the correlation. Compared with CON, at the phylum level, we found that *Verrucomicrobiota* was strongly correlated with most metabolites and *Synergistota* was negatively correlated with Tetradecanedioic acid (Figure 7A). At the genus level, a strong correlation between 12 differential genera and 24 differential metabolites at the genera level was observed in the CON and RF groups (Figure 7B). *Akkermansia* was negatively correlated with Chenodeoxycholic Acid and various amino acids, and was positively correlated with organic acid and its derivatives. The abundance of Pathogenic bacteria such as *Escherichia-Shigella* and *Actinomyces* decreased after inoculation with ruminal fluid and was associated with various amino acids, bile acids and other metabolites. *Rikenellaceae_RC9_gut_group* was negatively correlated with Trimethylamine-N-Oxide, and significantly positively correlated with the level of Chenodeoxycholic Acid and Lithocholic acid in serum. *Eubacterium_nodatum_group*, *Filifactor*, *Parabacteroides*, *Clostridiales_bacterium_canine_oral_taxon_082*, Porphyromonas and other genera were also correlated with many metabolites.

**Figure 8:**
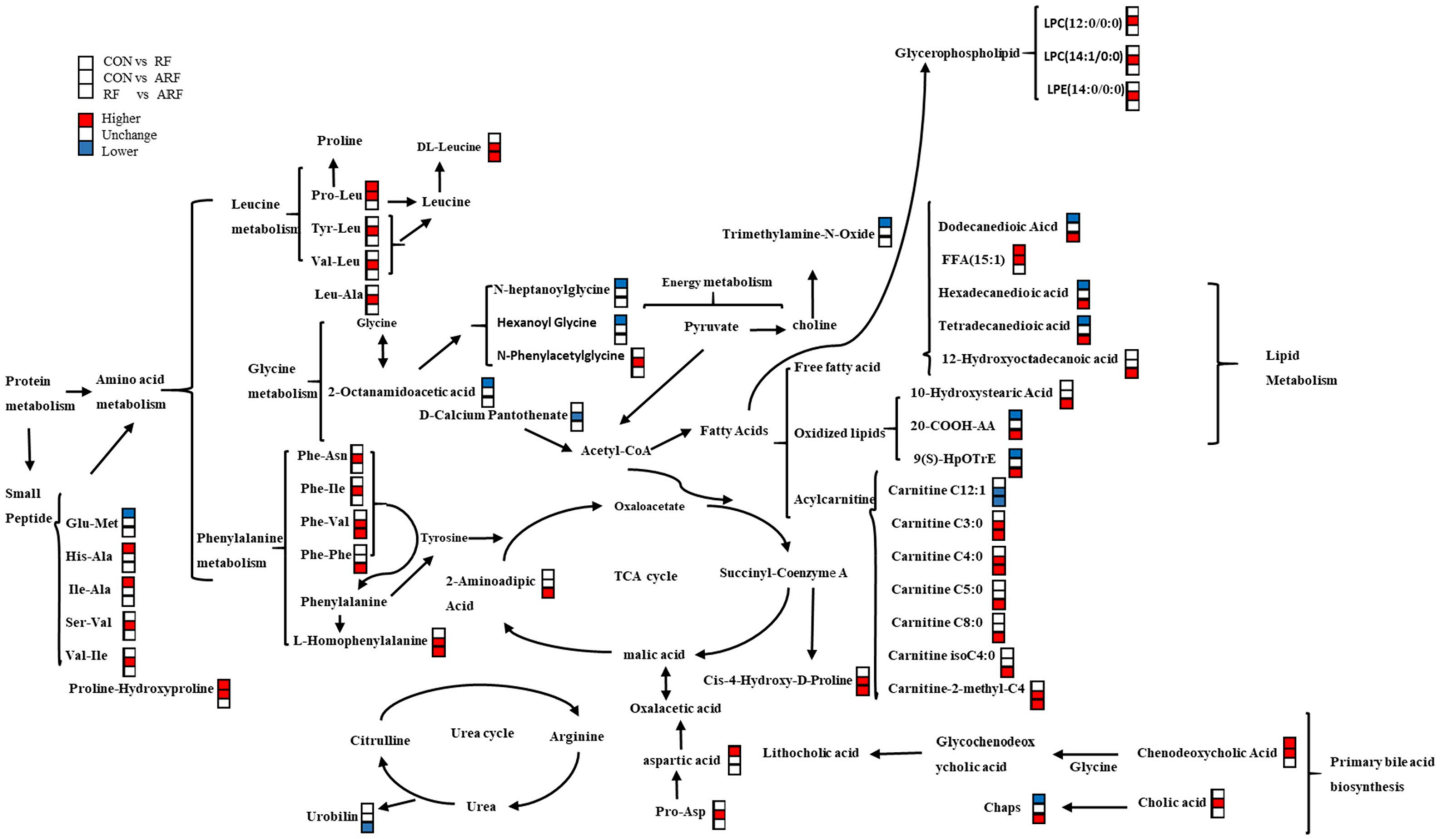
Correlation networks of fecal metabolites in CON, RF, and CON groups. Compared with the latter group, among the metabolites in the former group, the red symbol represents a significant increase, the white symbol represents no difference, and the blue symbol represents a decrease.

By taking the intersection of CON and ARF, 94 differential metabolites were obtained and narrowed down to 30 different metabolites (Figures 7C,D). At the phylum level, *Verrucomicrobiota* was negatively correlated with significantly different metabolites, *Fusobacteriota* was positively correlated with significantly different metabolites (Figure 7C). The change in *Akkermansia*, *Porphyromonas* and *NK4A214_group*, *Escherichia-Shigella* and *Parabacteroides* levels was associated with the serum levels of significantly different metabolites. Other microbiota were positively correlated with significantly different metabolites (Figure 7D). These results indicated that these differential microbiota were closely associated with, and might contribute to, the altered serum metabolic profiles in response to inoculation with ruminal fluid or autoclaved ruminal fluid. In order to better understand the metabolic regulation relationship, we have drawn a metabolic network diagram (Figure 8).

## 4. Discussion

### 4.1. Effect of ruminal fluid inoculation on weight of newborn lambs

Growth rate of lambs can potentially affect production performance in adulthood (Sato et al., 2010). Some early studies found that ruminal inoculation improved average daily gain (Pounden and Hibbs, 1949). A potential mechanism for such an effect was proposed to be a benefit of microorganism inoculation on amylase activity (Ziolecka et al., 1984). In contrast, other studies reported no significant improvements in weight gain due to ruminal fluid inoculation (De Barbieri et al., 2015). Although we did not detect a significant effect on weight gain over time, given that postpartum stress could lead to weight loss after birth in the newborn, the trend to promote a faster weight recovery in the RF group suggested a benefit of active microbial inoculation.

### 4.2. Effect of ruminal fluid inoculation on blood biochemical parameters

Blood parameters are important to evaluate the health status of animals in response to nutrition (Weingand et al., 1996). The lack of difference for most of the blood parameters related to liver and kidney function and inflammation suggested that ruminal fluid inoculation had no negative influence on the health of newborn lambs.

It is noteworthy that an increase of ALP, CHOL, and HDL was observed in the RF group. A greater concentration of ALP was observed in newborn infants experiencing active bone growth (Devyatkin et al., 2021). Thus, it is plausible to speculate that fresh ruminal fluid inoculation with active microorganisms was beneficial to the development of newborn lambs. CHOL is one of the important blood indices to estimate lipoprotein metabolism. A previous study found that multi-strain probiotic increased serum CHOL in neonatal dairy calves (Guo et al., 2022), which was similar to our study. However, a low blood concentration of CHOL was also observed in a previous study with spray-dried ruminal fluid inoculation (Rezai Sarteshnizi et al., 2020). Combined with the greater HDL blood content in RF relative to CON, the greater CHOL might be helpful in preventing lipid peroxidation and stimulating the synthesis of anti-inflammatory cytokines (such as interleukin-10) (Mertens and Holvoet, 2001). We speculated that active microorganism inoculation could potential regulate lipid metabolism and inflammatory reactions to influence the health of newborn lambs.

Overall, the blood biochemical parameters suggested that microorganism inoculation had no negative effect on health, and could potentially regulate lipid metabolism and inflammatory reactions.

### 4.3. The effect of ruminal fluid inoculation on gut microbiota

Gut microbiota is significantly different from the ruminal microbiota (Godoy-Vitorino et al., 2012), but data suggest that ruminal fluid inoculation still could regulate the gut microflora (Ji et al., 2018). In the present work, a distinct microbiota cluster of CON, RF and ARF was observed using PCA analysis, suggesting that ruminal fluid inoculation could influence the establishment of microflora in newborn lambs. The greater alpha-diversity index (Chao 1) in RF compared with CON suggested that ruminal fluid inoculation with active microorganisms could improve the diversity of the gut microbiota. A similar result was also observed in a previous study with goats (Abo-Donia et al., 2011).

The establishment of microbiota after birth might involve a sophisticated process (Wang et al., 2016). In the current study, we illustrated the change of gut microbial taxonomical composition with ruminal fluid inoculation at the phylum and genus levels in newborn lambs. In the ruminant, *Proteobacteria*, *Bacteroidetes* and *Firmicutes* were confirmed as the dominant phyla with important functions in maintaining health and production (Sadet et al., 2007). Therefore, our results with the greatest relative abundance of *Proteobacteria*, *Bacteroidetes* and *Firmicutes* suggested that ruminal fluid inoculation could accelerate the establishment of ruminal microbiota in newborn lambs. In our study, *Verrucomicrobia* was found at a lower proportion in lambs exclusively inoculated with ruminal fluid and were represented exclusively by the genus *Akkermansia*, in agreement with earlier studies (Saleem et al., 2012). As previously reported, the relative abundance of *Verrucomicrobia* was significantly higher in milk-fed lambs (Wang et al., 2016). The present study may indirectly suggest that ruminal fluid transplantation promotes the establishment of specific microbial population in lambs in order to better adapt to the transition from milk to solid feed. *Fusobacteria* is a phylum of anaerobic Gram-negative bacilli, with a shuttle-shaped morphology, commonly found in the oral cavity (Gupta and Sethi, 2014). The view that *Fusobacterium* is involved in animal infection within a certain range has long been accepted by many researchers (Roberts, 2000). Our results indicated that inoculation with ruminal fluid in lambs may also contribute to health by reducing the number of pathogenic bacteria. *Synergistota* is a bacterial phylum consisting of gram-negative anaerobes (Belibasakis et al., 2013). End-products of *Synergistota* are mainly acetate, propionate and isobutyrate (Kang et al., 2020). SCFA are the main source of energy for ruminants to maintain normal growth.

At the genus level, most of the *Rikenellaceae_RC9_gut_group* was upregulated. The *Rikenellaceae_RC9_gut_group* is the dominant bacteria in the intestine, associated with mucin degradation (Fan et al., 2020), and is significantly in a negative correlation with obesity (Arnoriaga-Rodríguez et al., 2020; Suzuki et al., 2020). It plays an important role in intestinal mucosal health. Moreover, the relative abundance of *Rikenellaceae_RC9_gut_group* was positively correlated with host feed utilization, volatile fatty acid and short-chain fatty acid metabolism (Derakhshani et al., 2017; Li et al., 2019) and significantly negatively correlated with the expression of inflammation-related immune genes such as insulin levels and interferon IFNγ (Gomez-Arango et al., 2016). *Parabacteroides* generate acetate to mitigate heparanase-exacerbated acute pancreatitis by reducing neutrophil infiltration (Lei et al., 2021). Acetate is the main SCFA. Some species of *Parabacteroides* Significant reduce in severity of intestinal inflammation in murine models of acute and chronic colitis through dextran sulfate sodium (Kverka et al., 2011). SCFA can regulate the function of multiple systems, such as the intestinal, neurological, endocrine and hematological systems, and there is considerable evidence that SCFAs play an important role in the maintenance of intestinal health and the prevention and improvement of many non-communicable diseases, including cancer (Doble et al., 2016). However, due to the interaction of microflora, the treatment in this study reduced the concentration of *Parabacteroides*.

In this study, most of the *Escherichia-Shigella* was significantly downregulated, *Escherichia-Shigella* is a conditional pathogenic bacteria (Qi et al., 2021). On the first day after birth, the rectum was invaded by *Escherichia-Shigella* (Alipour et al., 2018). Recent research suggests that the artificial feeding modal can increase the number of potential pathogens such as *Escherichia-Shigella* and slow the establishment of the anaerobic environment and anaerobic microorganism (Bi et al., 2019). *Actinomyces* can use carbohydrates to produce fatty acids and are classified as conditional pathogenic bacteria (Hall, 2008). *Eubacterium_nodatum_group* has the ability to Catabolic carbohydrates to SCFAs (mainly butyrate; Barcenilla et al., 2000). *Eubacterium_ nodatum_ Group* is common in oral cavity with periodontitis and other diseases, and is considered as pathogenic bacteria (Hill et al., 1987). The results varied a lot from one study to another, and there is no consistent conclusion about the *Akkermansia*. *Akkermansia*, produce SCFA by degrading mucin, are closely contacted with immunity (Ganesh et al., 2013). Increasing abundance of *Akkermansia muciniphila* could decreased body fat mass, increase glucose homeostasis, and lower adipose tissue inflammation (Everard et al., 2013; Dao et al., 2016). Recent research suggests that milk positively affected their growth (Wang et al., 2018). This may further prove that inoculation with ruminal fluid promotes the maturation of lamb microbes in order to better adapt to the transition from milk to solid feed. The previous studies have found that high-doses of heme iron intake significantly increased the abundance of *Akkermansia* in the intestinal tract of mice, but damaged the intestinal mucus layer (van Hylckama Vlieg et al., 2011). The results of the present study showed that inoculation with ruminal fluid could change bacteria of the Fusobacteria, *Akkermansia*, *Eubacterium_nodatum_group*, and the like. The effect was positively correlated with microbial activity in ruminal fluid. These results support the notion that active gut microbiota may inhibit the growth of pathogens, promote the maturation of growth-promoting microbes by altering the diversity and composition of the gut microbiota.

### 4.4. The effect of ruminal fluid inoculation on serum metabolites and correlations with serum metabolic profiles

Microbiota in the gut regulate metabolic reactions such as the production of bile acids, amino acid and fatty acids, which are essential for the health of the animal (Nicholson et al., 2012). In this study, the composition of serum metabolites in the RF group was different from the CON and ARF groups. Through the serum metabolomics analysis, it was concluded that the metabolism of bile acids (Chenodeoxycholic acid, Chaps, Lithocholic acid) was greatly influenced by feeding RF. Bile acids participate in the regulation of lipid and dextrose metabolism, and play a key role in regulating hepatic metabolic pathways (Hylemon et al., 2009). The bile acids may help control growth and composition of gut microbiota through FXR and TGR-5, which also helps protect the gut microbiota from inflammation (du et al., 2020). Previous research suggested that obeticholic acid, a derivative of chenodeoxycholic acid, can increase bile acid homeostasis, lower the expression of TNF-α, IL-6, and IL-1β, suggesting that bile acids alleviate inflammation (Xiong et al., 2017). Dietary chenodeoxycholic acid can improve growth performance and intestinal health by changing blood metabolism and intestinal bacteria in weaned piglets (Song et al., 2021). Lithocholic acid is formed by bacterial action of chenodeoxycholic acid salt and is usually combined with glycine or taurine (Bazzoli et al., 1982). Spearman correlation analysis between the affected metabolites showed that the elevated serum levels of chenodeoxycholic acid and Lithocholic acid were negatively correlated with *Escherichia-Shigella*, *Fusobacterium*, *Actinomyces*, *Porphyromonas*, *Eubacterium_ nodatum_ Group* and *Akkermansia*, and positively correlated with *Rikenellaceae_RC9_gut_group*. The results suggested that tumor gastric juice transplantation may improve the stability of the body by regulating the level of bile acid metabolism or regulating the composition and quantity of intestinal microbiota.

The end products of protein digestion in the digestive tract are often mostly small peptides rather than free amino acids, which are absorbed intact and enter the circulation as dipeptides or tripeptides (Rérat et al., 1988). Studies have shown that insufficient peptide in the ruminal fluid of dairy cows is the main factor limiting the growth of microorganisms, and small peptides are key factors for maximum growth efficiency of microorganisms (Brooks et al., 2012). Small peptides can promote the reproduction of beneficial microorganisms in the digestive tract and improve the synthesis of microbial proteins. Our results revealed an increasing trend of small peptides (such as proline-Hydroxyproline, His-Ala, Ile-Ala, and Pro-Leu), the correlation analysis showed that the elevated serum levels of small peptides was negatively correlated with *Escherichia-Shigella*, *Fusobacterium* and *Eubacterium_nodatum_group*. The tricarboxylic acid cycle is one of the most important energy metabolism pathways, our results on correlation network analysis showed that some small peptides may indirectly participate in the tricarboxylic acid cycle, i.e., enter the cycle after hydrolysis into free amino acids. The above results demonstrated that feeding RF promoted peptide absorption by enhancing the absorption rate of small peptides or change the absorbable amount of small peptides by reducing the abundance of *Escherichia Shigella* and other bacteria, thereby increasing the concentration of small peptides in the serum, thereby enhancing the metabolism of the body and promoting *Rikenellaceae_RC9_gut_group*. Xanthine and xanthoside are related to purine and pyrimidine metabolism, and the changes of these metabolites are related to microbial apoptosis (Fujihara and Shem, 2011; Wang et al., 2012). In this study, the contents of xanthine and xanthoside in serum of lambs increased, and were negatively correlated with *Escherichia Shigella*. Therefore, we speculate that the increase of xanthine and xanthoside content is related to the decrease of *Escherichia Shigella*.

When hepatic glycogen, a quick source of glucose for muscle, is depleted, adipose tissue lipolysis into free fatty acids provides these compounds to tissues as a source of energy (Cree-Green et al., 2017). The content in serum is very low under physiological condition, but the content increases abnormally in diabetes, severe liver dysfunction, hyperthyroidism and other diseases (Wolf et al., 2016). From the weight gain results, we can speculate that the feeding RF promoted feed digestion and nutrient absorption which could have favored liver glycogen synthesis and even glucose utilization for oxidation without the need to induce adipose tissue lipolysis. Free fatty acids are also one of the substances that contribute to oxidative stress (Jiang and Yan, 2022). Oxidized lipids are metabolites of polyunsaturated fatty acids (arachidonic acid, linoleic acid, alpha-linolenic acid) that undergo auto-oxidation or are generated by the action of specific enzymes (Tang et al., 2021). 20-COOH-AA is produced by a series of enzymatic oxidation reactions of arachidonic acid. Oxidative stress is a physiological state in which there is an imbalance between oxidation and antioxidant action in the body. Oxidized lipids serve as the material base in which unsaturated fatty acids are oxidized to form oxidative metabolites under oxidative stress, thus, affecting normal functions (Tsubone et al., 2019). Trimethylamine-N-Oxide is the promoter of atherosclerosis and participates in oxidative damage, endothelial dysfunction and other diseases (Moraes et al., 2015); Injury of vascular endothelial cells can be induced by increasing vascular oxidative stress, and mitochondrial dysfunction can also be induced by oxidative stress in mice (Li et al., 2018; Brunt et al., 2020). Plasma corticosterone is thought to be the main glucocorticoid involved in the regulation of the stress response in rodents (Mizobe et al., 1997). A striking association between serum corticosterone was observed during acute or repeated restraints, chronic unpredictable stress or heat stress (Gong et al., 2015). In the current study, the concentration of Free fatty acids, Oxidized lipids, Trimethylamine-N-Oxide and corticosterone in the RF group were downregulated compared with the other groups, and the blood biochemical indices showed that the concentration of HDL, which had the effect of antagonizing lipid peroxidation, was increased. It has been reported that probiotics can alleviate lipid accumulation, regulate blood lipid levels, increase the concentration of HDL-C, slow down the formation of oxidized lipids, and antagonize oxidative lipid damage in the liver (Nido et al., 2016). In our study, Free fatty acids, Oxidized lipids, Trimethylamine-N-Oxide, Corticosterone was negatively correlated with intestinal available bacteria such as *Rikenellaceae_RC9_gut_group*, and positively correlated with pathogenic bacteria such as *Escherichia Shigella*, *Actinomyces*, *Porphyromonas*. It has been pointed out that the disturbance of intestinal microbiota is one of the stressors leading to stress, and a stable microbiota can reduce the stress response of the body to a certain extent. Based on the above results, we speculate that the transplantation of rumen juice will promote the stabilization of intestinal microbiota, thereby reducing the levels of lipid oxide, trimethylamine oxide, cortisol, etc., to some extent, it can antagonize the stress response of lambs and improve the blood lipid level of lambs.

We found that RF treatment altered the levels of metabolites in lamb serum, with the metabolites differing in expression from the ARF group, and these changes were related to intestinal microorganisms. We speculate that the sterilized ruminal fluid was rich in protein, fatty acids and other nutrients, thus, promoting the development of the microbial community of the lambs and influencing the metabolites. The difference in the results of the combined RF and ARF groups suggested that active microorganisms in the ruminal were the main factors that contributed to the differences observed.

## 5. Conclusion

**Figure 9:**
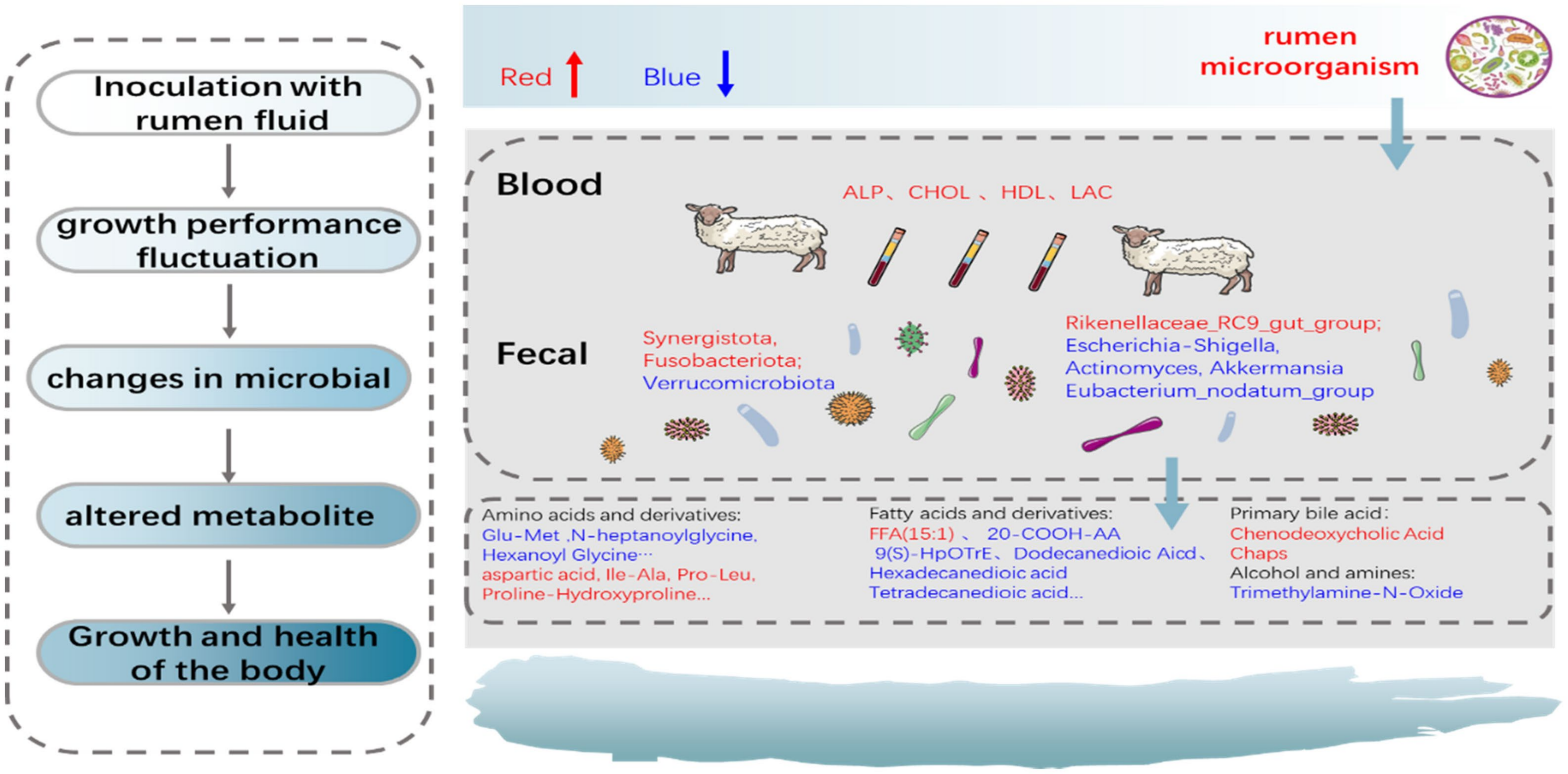
The proposed schematic diagram of the overall effects of the ruminal fluid inoculation on the gut microbial composition, function, and metabolites of lambs.

This study demonstrated that ruminal fluid inoculation with active microorganisms could accelerate weight recovery and maintain health in newborn lambs through regulating the establishment of gut microbiota. The proposed mechanism of the overall effects of the ruminal fluid inoculation on gut microbiota and metabolism is schematically represented in Figure 9. In general, this management strategy could be useful for promoting the development and alleviate the postpartum stress in newborn lambs.

## Data availability statement

The datasets presented in this study can be found in online repositories. The names of the repository/repositories and accession number(s) can be found at: NCBI - PRJNA923048; MetaboLights - MTBLS6998.

## Ethics statement

The animal study was reviewed and approved by Ethics Committee in Chongqing Academy of animal sciences (approval number: xky-20180716, 9 June 2021).

## Author contributions

LF, LW, and LL: data analysis and original draft writing. LZ, GW, and PZ: conducted the experiments and sample collection. JL and ZZ: draft reviewing. XD: experimental design, supervision, and original draft writing and reviewing. All authors contributed to the article and approved the submitted version.

## Funding

This study was supported by the National Natural Science Foundation of China (grant number 32002206, XD), Key Project of Chongqing Natural Science Foundation (grant number cstc2020jcyj–zdxmX0005, XD), the General Projects of Chongqing Natural Science Foundation (grant number cstc2021jcyj–msxmX1143, LL) and the Chongqing Performance Incentive Guide Special Project (20522, XD).

## Conflict of interest

The authors declare that the research was conducted in the absence of any commercial or financial relationships that could be construed as a potential conflict of interest.

## Publisher’s note

All claims expressed in this article are solely those of the authors and do not necessarily represent those of their affiliated organizations, or those of the publisher, the editors and the reviewers. Any product that may be evaluated in this article, or claim that may be made by its manufacturer, is not guaranteed or endorsed by the publisher.
