# Supplementary material for: Effects of inoculation with active microorganisms derived from adult goats on growth performance, gut microbiota and serum metabolome in newborn lambs
Source: Front Microbiol. 2023 Feb 13;14:1128271. doi: 10.3389/fmicb.2023.1128271 (PMC9969556; doi:10.3389/fmicb.2023.1128271)
Supplement: Supplementary file 1 [file Data_Sheet_1.docx]

**Supplementary Information**

**Table 1.** Growth performance during postnatal 15 days in lambs inoculated with rumen fluid (RF), autoclaved rumen fluid (ARF) and sterilized physiological saline (CON)

| Item | CON | RF | ARF | SEM | P |
| --- | --- | --- | --- | --- | --- |
| Day 1 (kg) | 2.30 | 2.25 | 2.22 | 0.17 | 0.89 |
| Day 3 (kg) | 2.26 | 2.20 | 2.12 | 0.17 | 0.73 |
| Day 6 (kg) | 2.16 | 2.15 | 2.06 | 0.17 | 0.80 |
| Day 9 (kg) | 2.12 | 2.12 | 2.04 | 0.16 | 0.83 |
| Day 12 (kg) | 2.14 | 2.13 | 2.03 | 0.15 | 0.70 |
| Day 15 (kg) | 2.11 | 2.17 | 2.07 | 0.14 | 0.80 |
| Total gain weight (kg) | -1.91 | -0.76 | -1.48 | 0.64 | 0.23 |
| Average daily gain weight (kg) | -0.13 | -0.05 | -0.10 | 0.04 | 0.21 |

**Table 2.** The concentration of serum metabolites in the CON vs RF

| Metabolites | P-value | Log2FC | VIP | UP/DOWN |
| --- | --- | --- | --- | --- |
| (R)-(-)-1-Amino-2-propanol | 5.90E-04 | -1.03E+00 | 1.92E+00 | down |
| (R)-(-)-Mandelic acid | 6.06E-02 | 2.44E+00 | 1.79E+00 | up |
| (R)-3-Hydroxymyristic acid | 1.11E-02 | -1.42E+00 | 1.61E+00 | down |
| (S)-2-Hydroxy-3-phenylpropanoic acid | 5.69E-02 | 1.56E+00 | 1.31E+00 | up |
| (S)-Leucic acid | 1.55E-01 | 3.96E+00 | 1.33E+00 | up |
| 1,4-Dihydro-1-Methyl-4-Oxo-3-Pyridinecarboxamide | 9.79E-02 | 4.46E+00 | 1.22E+00 | up |
| 1-Aminopropan-2-ol | 3.78E-04 | -2.52E+00 | 1.73E+00 | down |
| 2-Butyl-3-(4-hydroxybenzoyl)benzofuran | 2.13E-02 | -4.59E+00 | 1.97E+00 | down |
| 2-Hydroxy-2-Methyl Butyric acid | 6.45E-02 | 1.59E+00 | 1.61E+00 | up |
| 2-Hydroxy-3-Methyl Butanoic Acid | 6.40E-02 | 1.60E+00 | 1.63E+00 | up |
| 2-Hydroxyhexanoic acid | 1.55E-01 | 3.96E+00 | 1.33E+00 | up |
| 2-Hydroxyisocaproic Acid | 1.55E-01 | 3.96E+00 | 1.33E+00 | up |
| 2-Octanamidoacetic acid | 7.62E-02 | -2.28E+00 | 1.17E+00 | down |
| 20-COOH-AA | 1.53E-02 | -5.08E+00 | 2.19E+00 | down |
| 21-Deoxycortisol | 1.06E-03 | -3.01E+00 | 2.19E+00 | down |
| 3,5-Dimethoxy-4-Hydroxycinnamic Acid | 1.87E-02 | 5.00E+00 | 2.22E+00 | up |
| 3-Hydroxy-tetradecanoic acid | 1.11E-02 | -1.42E+00 | 1.61E+00 | down |
| 3-hydroxyphenylacetic acid | 5.90E-02 | 2.58E+00 | 1.85E+00 | up |
| 4-Methylhippuric Acid | 2.43E-02 | 1.32E+00 | 1.66E+00 | up |
| 4-acetoxyphenol | 1.09E-01 | 1.06E+00 | 1.18E+00 | up |
| 5-Hydroxyhexanoic Acid | 1.55E-01 | 3.96E+00 | 1.33E+00 | up |
| 8-Azaguanine | 1.00E-01 | 4.49E+00 | 1.24E+00 | up |
| 9(S)-HpOTrE | 2.95E-02 | -3.58E+00 | 1.81E+00 | down |
| Biliverdin | 9.34E-03 | -1.47E+00 | 1.70E+00 | down |
| Chaps | 3.73E-05 | -3.21E+00 | 2.15E+00 | down |
| Chenodeoxycholic Acid | 1.37E-04 | 3.11E+00 | 2.27E+00 | up |
| Corticosterone | 2.93E-03 | -1.07E+00 | 1.49E+00 | down |
| Daidzein | 5.82E-03 | 1.26E+00 | 1.93E+00 | up |
| Dodecanedioic Aicd | 1.83E-02 | -1.34E+00 | 1.73E+00 | down |
| FFA(15:1) | 4.56E-03 | 1.08E+00 | 1.73E+00 | up |
| Glu-Met | 8.20E-03 | -1.24E+00 | 1.85E+00 | down |
| Hexadecanedioic acid | 4.36E-02 | -2.64E+00 | 1.64E+00 | down |
| Hexanoyl Glycine | 6.90E-02 | -1.44E+00 | 1.21E+00 | down |
| His-Ala | 9.96E-02 | 1.11E+00 | 1.11E+00 | up |
| Ile-Ala | 3.94E-02 | 1.23E+00 | 1.42E+00 | up |
| Iminodiacetic acid | 1.66E-03 | -2.04E+00 | 1.93E+00 | down |
| Isohomovanillic acid | 3.90E-02 | 1.20E+00 | 1.72E+00 | up |
| Lithocholic acid | 1.41E-04 | 2.36E+00 | 1.47E+00 | up |
| N'-Methyl-2-pyridone-5-carboxamide | 1.01E-01 | 4.48E+00 | 1.28E+00 | up |
| N-heptanoylglycine | 1.01E-01 | -1.71E+00 | 1.07E+00 | down |
| Octadecanedioic acid | 1.66E-02 | -2.43E+00 | 1.64E+00 | down |
| Oxypurinol | 1.00E-01 | 4.49E+00 | 1.24E+00 | up |
| Phenyllactate(Pla) | 5.53E-02 | 1.70E+00 | 1.37E+00 | up |
| Pro-Leu | 1.32E-02 | 1.36E+00 | 1.60E+00 | up |
| Proline-Hydroxyproline | 5.86E-03 | 1.51E+00 | 1.94E+00 | up |
| Tetradecanedioic acid | 2.93E-02 | -2.58E+00 | 1.82E+00 | down |
| Trimethylamine-N-Oxide | 3.78E-04 | -2.52E+00 | 1.73E+00 | down |
| Tropine | 5.53E-02 | 1.70E+00 | 1.37E+00 | up |
| Urobilin | 1.10E-01 | -4.29E+00 | 1.21E+00 | down |
| Xanthine | 1.22E-01 | 1.01E+00 | 1.11E+00 | up |
| Xanthosine | 1.05E-01 | 4.39E+00 | 1.21E+00 | up |

**Table 3.** The concentration of serum metabolites in the CON vs ARF

| Metabolites | P-value | Log2FC | VIP | UP/DOWN |
| --- | --- | --- | --- | --- |
| (R)-(-)-1-Amino-2-propanol | 4.32E-08 | 1.43E+00 | 1.93E+00 | up |
| (R)-(-)-Mandelic acid | 2.87E-03 | 2.84E+00 | 1.77E+00 | up |
| (R)-3-Hydroxymyristic acid | 2.85E-02 | -1.04E+00 | 1.06E+00 | down |
| (S)-2-Hydroxy-3-phenylpropanoic acid | 2.78E-03 | 2.04E+00 | 1.67E+00 | up |
| (S)-Leucic acid | 5.22E-02 | 3.38E+00 | 1.51E+00 | up |
| 1,4-Dihydro-1-Methyl-4-Oxo-3-Pyridinecarboxamide | 1.06E-03 | 5.65E+00 | 1.81E+00 | up |
| 2-Amino-5-methyl-4-phenylthiazole | 1.75E-03 | 1.61E+00 | 1.68E+00 | up |
| 2-Aminoethanesulfinic Acid | 1.11E-02 | -1.71E+00 | 1.43E+00 | down |
| 2-Hydroxy-2-Methyl Butyric acid | 4.32E-02 | 1.72E+00 | 1.36E+00 | up |
| 2-Hydroxy-3-Methyl Butanoic Acid | 4.37E-02 | 1.74E+00 | 1.37E+00 | up |
| 2-Hydroxyhexanoic acid | 5.22E-02 | 3.38E+00 | 1.51E+00 | up |
| 2-Hydroxyisocaproic Acid | 5.22E-02 | 3.38E+00 | 1.51E+00 | up |
| 2-Methyl-1-Pyrroline | 1.44E-02 | 1.02E+00 | 1.50E+00 | up |
| 2-Methylhippuric acid | 3.62E-05 | 1.02E+00 | 1.59E+00 | up |
| 3,5-Dimethoxy-4-Hydroxycinnamic Acid | 1.09E-03 | 5.92E+00 | 1.89E+00 | up |
| 3-Amino-4-Hydroxybenzoic Acid | 4.20E-03 | 1.56E+00 | 1.50E+00 | up |
| 3-Amino-5-hydroxybenzoic acid | 4.20E-03 | 1.56E+00 | 1.50E+00 | up |
| 3-Epideoxycholic acid | 1.05E-02 | 1.09E+00 | 1.31E+00 | up |
| 3-Hydroxy-tetradecanoic acid | 2.85E-02 | -1.04E+00 | 1.06E+00 | down |
| 3-Hydroxyanthranilic Acid | 4.20E-03 | 1.56E+00 | 1.50E+00 | up |
| 3-Indolepropionic Acid | 2.61E-05 | 1.05E+00 | 1.52E+00 | up |
| 3-hydroxyphenylacetic acid | 4.66E-03 | 2.95E+00 | 1.74E+00 | up |
| 4-Acetylaminobenzoic acid | 2.05E-06 | 1.40E+00 | 1.92E+00 | up |
| 4-Methylhippuric Acid | 5.41E-04 | 1.94E+00 | 1.43E+00 | up |
| 4-acetoxyphenol | 1.30E-03 | 1.76E+00 | 1.75E+00 | up |
| 5,6-Dihydroxyindole-2-Carboxylic Acid | 3.21E-03 | 1.09E+00 | 1.53E+00 | up |
| 5-Aminoimidazole ribonucleotide | 1.04E-01 | -1.19E+00 | 1.37E+00 | down |
| 5-Hydroxyhexanoic Acid | 5.22E-02 | 3.38E+00 | 1.51E+00 | up |
| 5-Hydroxyindole-3-Acetic Acid | 5.96E-04 | 1.05E+00 | 1.74E+00 | up |
| 6-Aminocaproic-Acid | 5.77E-04 | 2.26E+00 | 1.62E+00 | up |
| 8-Azaguanine | 1.04E-03 | 5.63E+00 | 1.80E+00 | up |
| Aminolevulinic acid | 5.77E-04 | 2.26E+00 | 1.62E+00 | up |
| Arachidyl-glycine | 9.77E-03 | 3.58E+00 | 1.81E+00 | up |
| Biotin | 3.49E-02 | 1.20E+00 | 1.36E+00 | up |
| Caffeic Acid | 1.41E-05 | 1.02E+00 | 1.91E+00 | up |
| Carnitine C12:1 | 1.72E-02 | -4.10E+00 | 1.82E+00 | down |
| Carnitine C3:0 | 3.71E-03 | 1.41E+00 | 1.69E+00 | up |
| Carnitine C4:0 | 7.05E-03 | 1.39E+00 | 1.69E+00 | up |
| Carnitine isoC4:0 | 7.05E-03 | 1.39E+00 | 1.69E+00 | up |
| Chenodeoxycholic Acid | 2.72E-03 | 2.97E+00 | 1.73E+00 | up |
| Cholic acid | 6.69E-02 | 3.50E+00 | 1.12E+00 | up |
| Cis-4-Hydroxy-D-Proline | 5.77E-04 | 2.26E+00 | 1.62E+00 | up |
| Cis-L-3-hydroxyproline | 5.77E-04 | 2.26E+00 | 1.62E+00 | up |
| D-Calcium Pantothenate | 1.26E-03 | -2.21E+00 | 1.47E+00 | down |
| DL-Leucine | 5.77E-04 | 2.26E+00 | 1.62E+00 | up |
| Daidzein | 2.66E-02 | 1.60E+00 | 1.39E+00 | up |
| Dopaquinone | 9.83E-03 | 3.27E+00 | 1.49E+00 | up |
| Ethionamide | 1.80E-03 | 1.00E+00 | 1.67E+00 | up |
| Ethylsalicylate | 1.34E-03 | 1.06E+00 | 1.72E+00 | up |
| FFA(15:1) | 4.53E-02 | 1.21E+00 | 1.19E+00 | up |
| Gamma-Mercholic Acid | 6.69E-02 | 3.50E+00 | 1.12E+00 | up |
| Hippuric Acid | 5.71E-07 | 1.46E+00 | 1.93E+00 | up |
| His-Ala | 8.16E-02 | 1.44E+00 | 1.01E+00 | up |
| Hydroxyphenyllactic acid | 5.18E-04 | 1.03E+00 | 1.81E+00 | up |
| Iminodiacetic acid | 2.05E-02 | -1.56E+00 | 1.59E+00 | down |
| Isohomovanillic acid | 1.78E-02 | 1.38E+00 | 1.40E+00 | up |
| L-Homophenylalanine | 6.06E-03 | 2.01E+00 | 1.64E+00 | up |
| L-Methionine sulfoxide | 1.14E-08 | 1.07E+00 | 1.97E+00 | up |
| LPC(12:0/0:0) | 7.54E-03 | 1.52E+00 | 1.58E+00 | up |
| LPC(14:1/0:0) | 4.02E-03 | 1.24E+00 | 1.63E+00 | up |
| LPE(14:0/0:0) | 5.50E-04 | 1.29E+00 | 1.63E+00 | up |
| Leu-Ala | 1.84E-02 | 1.04E+00 | 1.28E+00 | up |
| Lythramine | 1.24E-01 | 1.65E+00 | 1.27E+00 | up |
| N'-Methyl-2-pyridone-5-carboxamide | 8.56E-04 | 5.60E+00 | 1.82E+00 | up |
| N-Cinnamylglycine | 2.50E-03 | 1.55E+00 | 1.59E+00 | up |
| N-Formylmethionine | 1.36E-02 | 1.30E+00 | 1.43E+00 | up |
| N-Phenylacetylglycine | 3.62E-05 | 1.02E+00 | 1.59E+00 | up |
| Oxypurinol | 1.04E-03 | 5.63E+00 | 1.80E+00 | up |
| Phe-Asn | 2.33E-02 | 1.16E+00 | 1.32E+00 | up |
| Phe-Ile | 1.56E-02 | 1.09E+00 | 1.35E+00 | up |
| Phe-Val | 1.15E-03 | 1.07E+00 | 1.60E+00 | up |
| Phenylacetyl-L-Glutamine | 5.66E-04 | 1.57E+00 | 1.75E+00 | up |
| Phenyllactate(Pla) | 3.03E-03 | 2.17E+00 | 1.66E+00 | up |
| Piperic acid | 1.26E-03 | 1.01E+00 | 1.68E+00 | up |
| Porphobilinogen | 1.19E-03 | 1.02E+00 | 1.70E+00 | up |
| Pro-Asp | 1.51E-03 | 1.06E+00 | 1.52E+00 | up |
| Pro-Leu | 1.31E-03 | 1.31E+00 | 1.39E+00 | up |
| Proline-Hydroxyproline | 7.37E-04 | 1.47E+00 | 1.68E+00 | up |
| S-Allyl-L-cysteine | 3.01E-03 | -1.86E+00 | 1.41E+00 | down |
| ST-638 | 6.87E-02 | 3.50E+00 | 1.01E+00 | up |
| Salicylaldehyde | 3.92E-07 | 1.34E+00 | 1.92E+00 | up |
| Ser-Val | 1.18E-03 | 1.00E+00 | 1.52E+00 | up |
| Serotonin | 1.48E-02 | 1.28E+00 | 1.44E+00 | up |
| Shikimic Acid | 8.71E-02 | 2.38E+00 | 1.12E+00 | up |
| Trans-4-Hydroxy-L-Proline | 5.77E-04 | 2.26E+00 | 1.62E+00 | up |
| Tropine | 3.03E-03 | 2.17E+00 | 1.66E+00 | up |
| Tyr-Leu | 2.58E-02 | 1.64E+00 | 1.26E+00 | up |
| Urobilin | 1.01E-01 | -5.57E+00 | 1.57E+00 | down |
| Val-Ile | 1.10E-02 | 1.22E+00 | 1.16E+00 | up |
| Val-Leu | 1.10E-02 | 1.22E+00 | 1.16E+00 | up |
| Xanthine | 1.28E-03 | 1.68E+00 | 1.73E+00 | up |
| Xanthosine | 8.26E-04 | 5.55E+00 | 1.81E+00 | up |
| alpha-Muricholic acid | 6.69E-02 | 3.50E+00 | 1.12E+00 | up |
| beta-Muricholic acid | 6.69E-02 | 3.50E+00 | 1.12E+00 | up |

**Table 4.** The concentration of serum metabolites in the RF vs ARF

| Metabolites | P-value | Log2FC | VIP | UP/DOWN |
| --- | --- | --- | --- | --- |
| (R)-(-)-1-Amino-2-propanol | 2.49E-09 | 2.47E+00 | 2.16E+00 | up |
| 1,4-Dihydro-1-Methyl-4-Oxo-3-Pyridinecarboxamide | 7.02E-02 | 1.19E+00 | 1.19E+00 | up |
| 10-Hydroxystearic Acid | 1.55E-02 | 1.04E+00 | 1.46E+00 | up |
| 12-Hydroxyoctadecanoic acid | 5.22E-02 | 1.28E+00 | 1.41E+00 | up |
| 2-Aminoadipic Acid | 1.74E-02 | 1.02E+00 | 1.56E+00 | up |
| 2-Aminoethanesulfinic Acid | 5.34E-04 | -2.07E+00 | 1.63E+00 | down |
| 2-Butyl-3-(4-hydroxybenzoyl)benzofuran | 3.46E-03 | 3.76E+00 | 1.82E+00 | up |
| 20-COOH-AA | 2.34E-03 | 4.45E+00 | 2.02E+00 | up |
| 21-Deoxycortisol | 6.57E-03 | 3.07E+00 | 1.97E+00 | up |
| 3-Epideoxycholic acid | 2.64E-03 | 1.60E+00 | 1.86E+00 | up |
| 4-Acetylaminobenzoic acid | 7.12E-07 | 1.49E+00 | 2.13E+00 | up |
| 5-Aminoimidazole ribonucleotide | 1.87E-01 | -1.13E+00 | 1.48E+00 | down |
| 5-Hydroxy-L-Tryptophan | 2.85E-03 | 1.32E+00 | 1.06E+00 | up |
| 6-Aminocaproic-Acid | 8.14E-06 | 3.47E+00 | 2.04E+00 | up |
| 8-Azaguanine | 8.45E-02 | 1.14E+00 | 1.17E+00 | up |
| 9(S)-HpOTrE | 6.37E-03 | 2.85E+00 | 1.70E+00 | up |
| Aminolevulinic acid | 8.14E-06 | 3.47E+00 | 2.04E+00 | up |
| Arachidyl-glycine | 1.20E-02 | 3.12E+00 | 1.98E+00 | up |
| BQ-123 free state | 1.90E-02 | -1.40E+00 | 1.51E+00 | down |
| Caffeic Acid | 8.53E-06 | 1.03E+00 | 2.09E+00 | up |
| Carnitine C12:1 | 2.78E-05 | -3.41E+00 | 2.01E+00 | down |
| Carnitine C3:0 | 5.10E-03 | 1.28E+00 | 1.84E+00 | up |
| Carnitine C4:0 | 8.68E-03 | 1.30E+00 | 1.86E+00 | up |
| Carnitine C5:0 | 2.13E-03 | 1.10E+00 | 1.93E+00 | up |
| Carnitine C8:0 | 9.31E-03 | 1.10E+00 | 1.73E+00 | up |
| Carnitine isoC4:0 | 8.68E-03 | 1.30E+00 | 1.86E+00 | up |
| Carnitine-2-methyl-C4 | 2.13E-03 | 1.10E+00 | 1.93E+00 | up |
| Chaps | 8.84E-04 | 2.64E+00 | 1.98E+00 | up |
| Cis-4-Hydroxy-D-Proline | 8.14E-06 | 3.47E+00 | 2.04E+00 | up |
| Cis-L-3-hydroxyproline | 8.14E-06 | 3.47E+00 | 2.04E+00 | up |
| D-Calcium Pantothenate | 9.89E-04 | -2.16E+00 | 1.63E+00 | down |
| DL-Leucine | 8.14E-06 | 3.47E+00 | 2.04E+00 | up |
| Dodecanedioic Aicd | 9.54E-04 | 1.53E+00 | 1.92E+00 | up |
| Dopaquinone | 8.63E-03 | 3.68E+00 | 1.79E+00 | up |
| Hexadecanedioic acid | 1.50E-02 | 2.37E+00 | 1.65E+00 | up |
| Hippuric Acid | 3.41E-07 | 1.47E+00 | 2.09E+00 | up |
| Indoxylsulfuric acid | 4.44E-02 | 1.17E+00 | 1.09E+00 | up |
| L-Homophenylalanine | 3.57E-03 | 2.54E+00 | 1.86E+00 | up |
| Lithocholic acid | 1.57E-03 | -1.32E+00 | 1.75E+00 | down |
| N'-Methyl-2-pyridone-5-carboxamide | 8.75E-02 | 1.12E+00 | 1.18E+00 | up |
| N-Cinnamylglycine | 1.08E-03 | 2.10E+00 | 1.98E+00 | up |
| N-Methyl-L-Glutamate | 1.74E-02 | 1.02E+00 | 1.56E+00 | up |
| Octadecanedioic acid | 1.69E-02 | 1.99E+00 | 1.55E+00 | up |
| Oxypurinol | 8.45E-02 | 1.14E+00 | 1.17E+00 | up |
| Phe-Asn | 4.73E-02 | 1.38E+00 | 1.49E+00 | up |
| Phe-Phe | 3.17E-02 | 1.49E+00 | 1.51E+00 | up |
| Phenylacetyl-L-Glutamine | 4.28E-04 | 1.81E+00 | 2.10E+00 | up |
| S-Allyl-L-cysteine | 2.72E-02 | -1.58E+00 | 1.54E+00 | down |
| Salicylaldehyde | 3.01E-07 | 1.32E+00 | 2.12E+00 | up |
| Sebacate | 1.37E-03 | 1.11E+00 | 1.62E+00 | up |
| Shikimic Acid | 8.49E-02 | 2.46E+00 | 1.36E+00 | up |
| Tetradecanedioic acid | 1.26E-02 | 2.13E+00 | 1.73E+00 | up |
| Trans-4-Hydroxy-L-Proline | 8.14E-06 | 3.47E+00 | 2.04E+00 | up |
| Urobilin | 2.63E-01 | -1.28E+00 | 1.29E+00 | down |
| Xanthosine | 7.65E-02 | 1.16E+00 | 1.19E+00 | up |
| alpha-hexylcinnamaldehyde | 1.58E-02 | 1.65E+00 | 1.71E+00 | up |
| α-CEHC | 3.40E-04 | -1.05E+00 | 1.69E+00 | down |


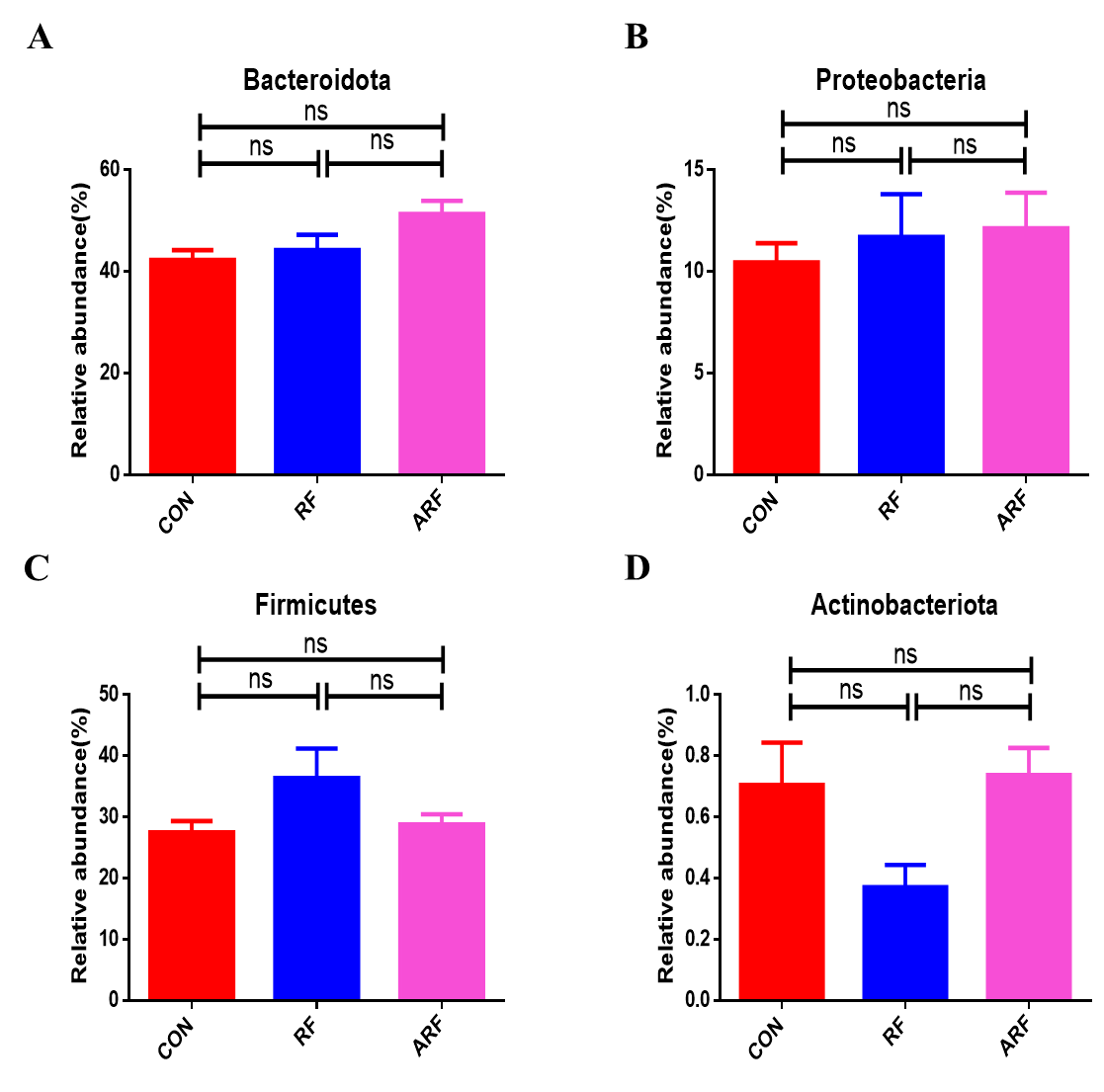


Figure 1. Relative abundance of microbiota at phylum level. (A) Relative abundance of Bacteroidota in the three groups. (B) Relative abundanceRelative abundance of Proteobacteria in the three groups. (C) Relative abundance of Firmicutes in the three groups. (D) Relative abundance of Actinobacteriota in the three groups. n = 8, ns indicates no significance.


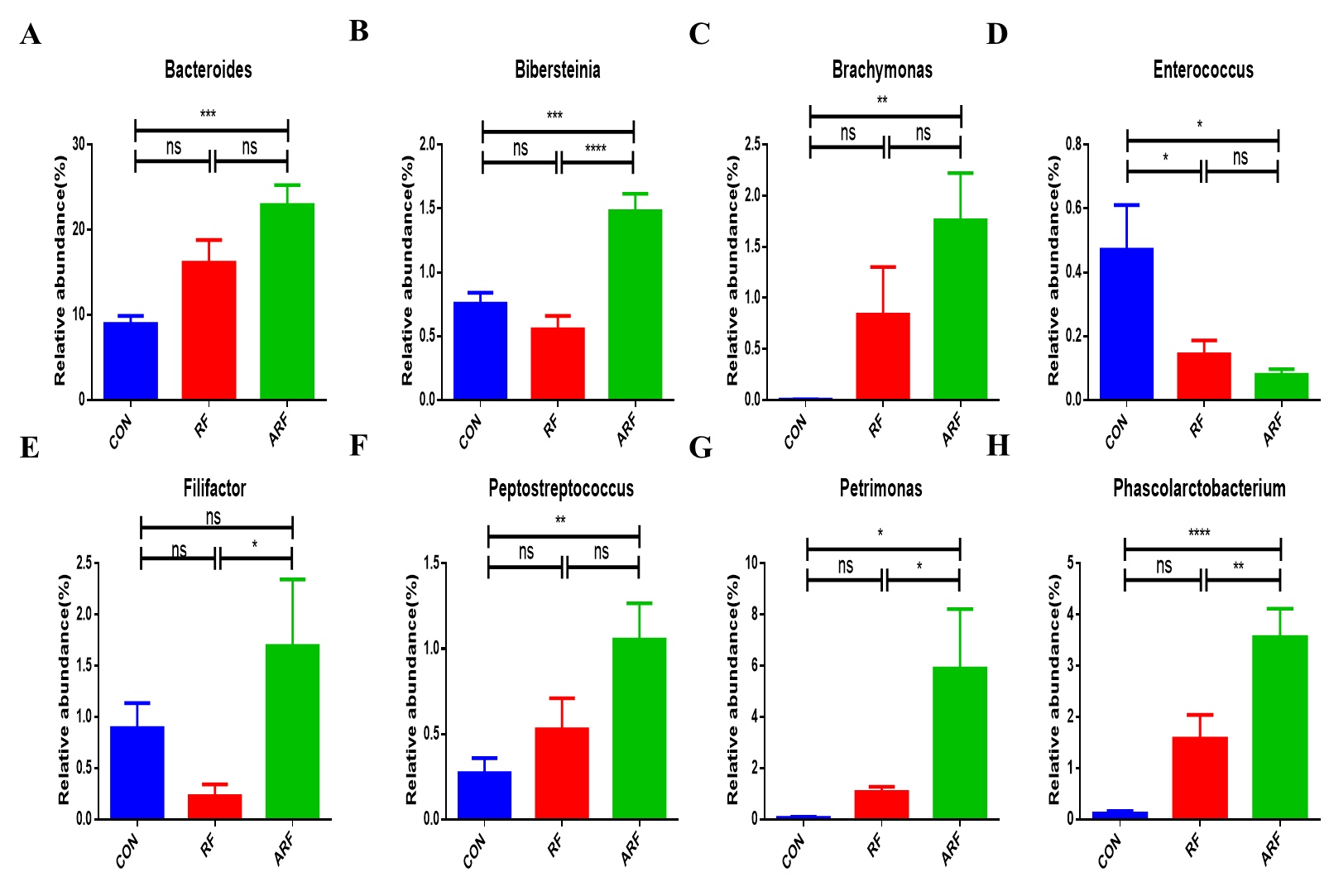


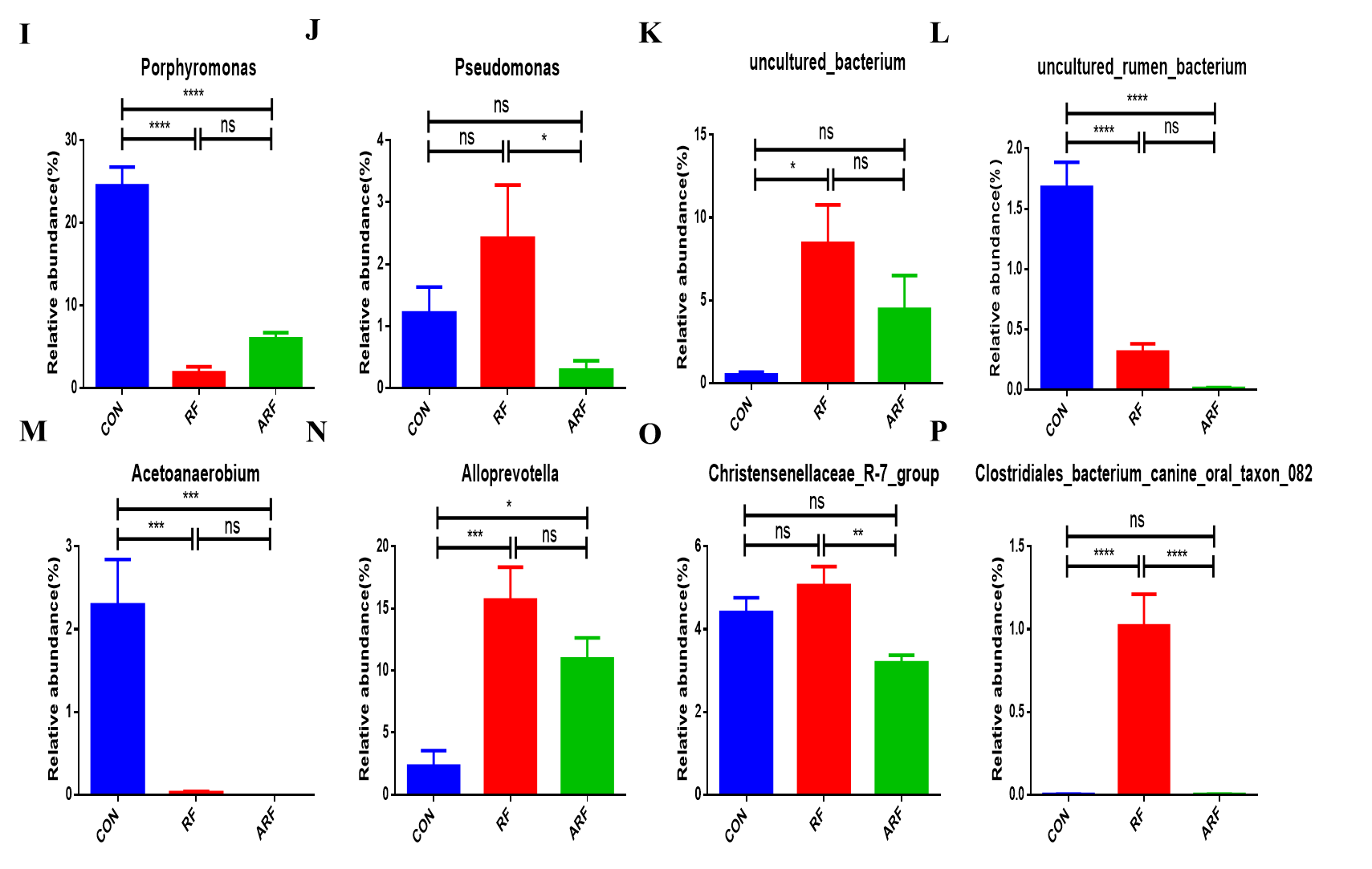


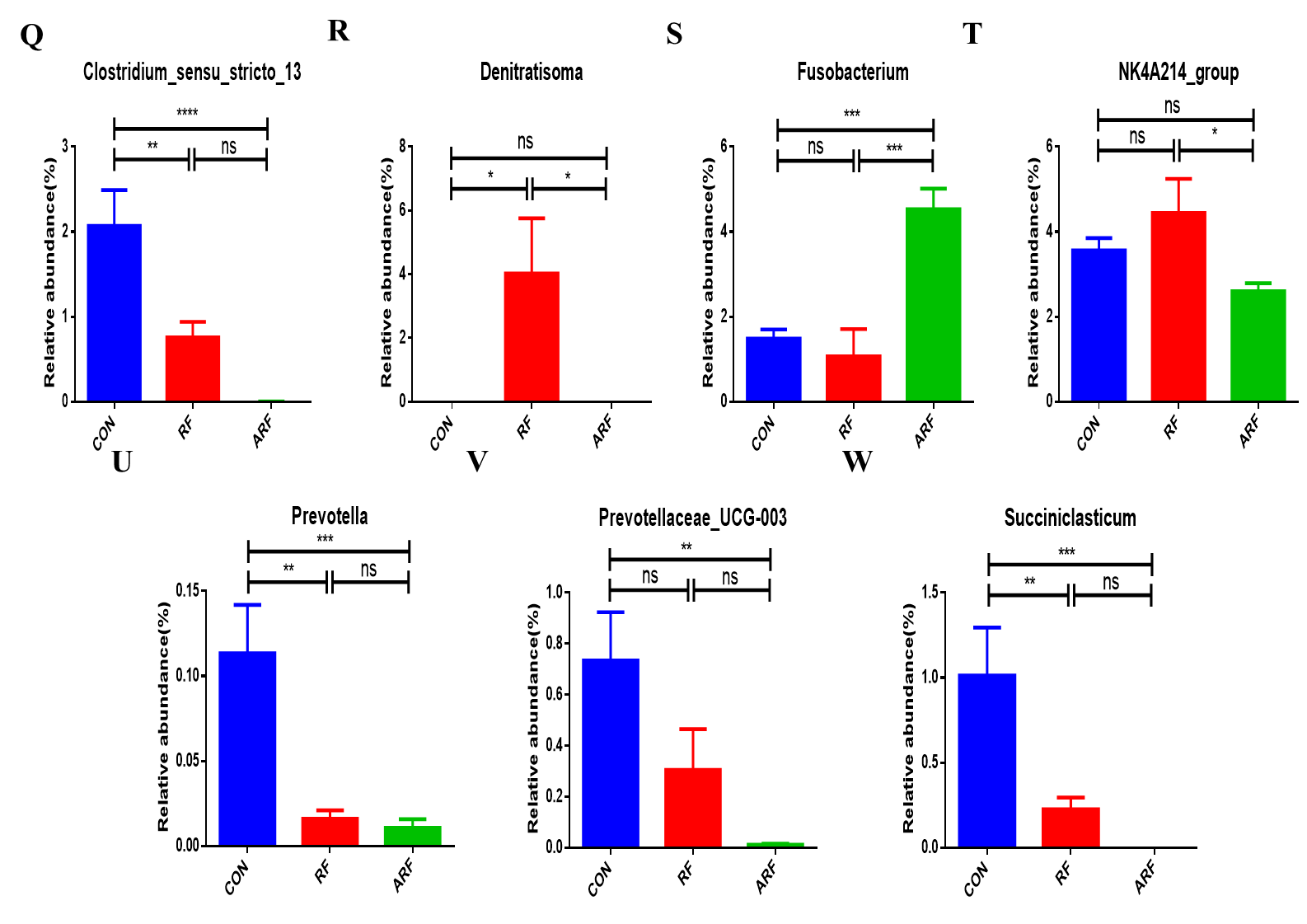


Figure 2. Relative abundance of microbiota at the genera level. (A) Relative abundance of Bacteroides in the three groups. (B) Relative abundance of Bibersteinia in the three groups. (C) Relative abundance of Brachymonas in the three groups. (D) Relative abundance of Enterococcus in the three groups. (E) Relative abundance of Filifactor in the three groups. (F) Relative abundance of Peptostreptococcus in the three groups. (G) Relative abundance of Petrimonas in the three groups. (H) Relative abundance of Phascolarctobacterium in the three groups. (I) Relative abundance of Porphyromonas in the three groups. (J) Relative abundance of Pseudomonas in the three groups. (K) Relative abundance of uncultured_bacterium in the three groups. (L) Relative abundance of uncultured_rumen_bacterium in the three groups. (M) Relative abundance of Acetoanaerobium in the three groups. (N) Relative abundance of Alloprevotella in the three groups. (O) Relative abundance of Christensenellaceae_R-7_group in the three groups. (P) Relative abundance of Clostridiales_bacterium_canine_oral_taxon_082 in the three groups. (Q) Relative abundance of Clostridium_sensu_stricto_13 in the three groups. (R) Relative abundance of Denitratisoma in the three groups. (S) Relative abundance of Fusobacterium in the three groups. (T) Relative abundance of NK4A214_group in the three groups. (U) Relative abundance of Prevotella in the three groups. (V) Relative abundance of Prevotellaceae_UCG-003 in the three groups. (W) Ratio of Succiniclasticum in the three groups. n = 8, * p < 0.05, ** p < 0.01, *** p < 0.001, **** p < 0.0001, ns indicates no significance.


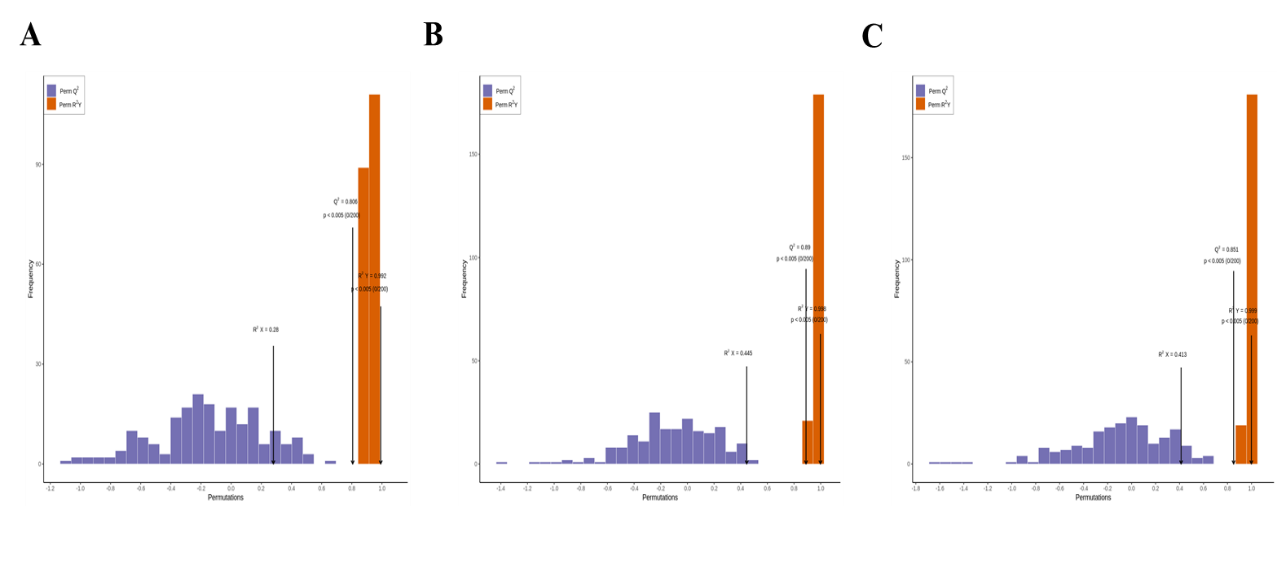


Figure 3. Metabolomic analysis of permutation test of OPLS-DA. (A) Permutation test of the OPLS-DA model for the CON vs RF. (B) Permutation test of the OPLS-DA model for the CON vs ARF. (C) Permutation test of the OPLS-DA model for the RF vs ARF.
